# Searching for Signaling Balance through the Identification of Genetic Interactors of the Rab Guanine-Nucleotide Dissociation Inhibitor *gdi-1*

**DOI:** 10.1371/journal.pone.0010624

**Published:** 2010-05-13

**Authors:** Anna Y. Lee, Richard Perreault, Sharon Harel, Elodie L. Boulier, Matthew Suderman, Michael Hallett, Sarah Jenna

**Affiliations:** 1 McGill Centre for Bioinformatics, McGill University, Montréal, Québec, Canada; 2 School of Computer Science, McGill University, Montréal, Québec, Canada; 3 Rosalind and Morris Goodman Cancer Centre, McGill University, Montréal, Québec, Canada; 4 Department of Chemistry, Université du Québec à Montréal, Montréal, Québec, Canada; 5 Pharmaqam, Université du Québec à Montréal, Montréal, Québec, Canada; 6 Biomed, Université du Québec à Montréal, Montréal, Québec, Canada; Deutsches Krebsforschungszentrum, Germany

## Abstract

**Background:**

The symptoms of numerous diseases result from genetic mutations that disrupt the homeostasis maintained by the appropriate integration of signaling gene activities. The relationships between signaling genes suggest avenues through which homeostasis can be restored and disease symptoms subsequently reduced. Specifically, disease symptoms caused by loss-of-function mutations in a particular gene may be reduced by concomitant perturbations in genes with antagonistic activities.

**Methodology/Principal Findings:**

Here we use network-neighborhood analyses to predict genetic interactions in *Caenorhabditis elegans* towards mapping antagonisms and synergisms between genes in an animal model. Most of the predicted interactions are novel, and the experimental validation establishes that our approach provides a gain in accuracy compared to previous efforts. In particular, we identified genetic interactors of *gdi-1*, the orthologue of *GDI1*, a gene associated with mental retardation in human. Interestingly, some *gdi-1* interactors have human orthologues with known neurological functions, and upon validation of the interactions in mammalian systems, these orthologues would be potential therapeutic targets for *GDI1*-associated neurological disorders. We also observed the conservation of a *gdi-1* interaction between different cellular systems in *C. elegans*, suggesting the involvement of *GDI1* in human muscle degeneration.

**Conclusions/Significance:**

We developed a novel predictor of genetic interactions that may have the ability to significantly streamline the identification of therapeutic targets for monogenic disorders involving genes conserved between human and *C. elegans*.

## Introduction

Many biological mechanisms depend on a state of signaling homeostasis maintained by the appropriate integration of the synergistic and antagonistic activities of signaling genes [1]. Accordingly, the symptoms of numerous diseases result from genetic mutations that disrupt this homeostasis [2]–[5]. The relationships between signaling genes suggest avenues through which homeostasis can be restored and disease symptoms subsequently reduced. Specifically, disruptions caused by loss-of-function mutations in a particular gene may be compensated by concomitant perturbations in genes with antagonistic activities.

Antagonisms and synergisms between genes can be identified via genetic interactions. A genetic interaction between two genes exists when the phenotypic effect of a perturbation (e.g. mutation, RNAi treatment, drug targeting) in one gene is dependent upon a perturbation in the other gene. Thus, disease symptoms caused by mutations in a given gene may be compensated by perturbing genetic interactors of the gene. That is, the genetic interactors are potential therapeutic targets. Therefore, the identification of genetic interactions is an important step towards the development of treatments for monogenic disorders.

**Figure 1:**
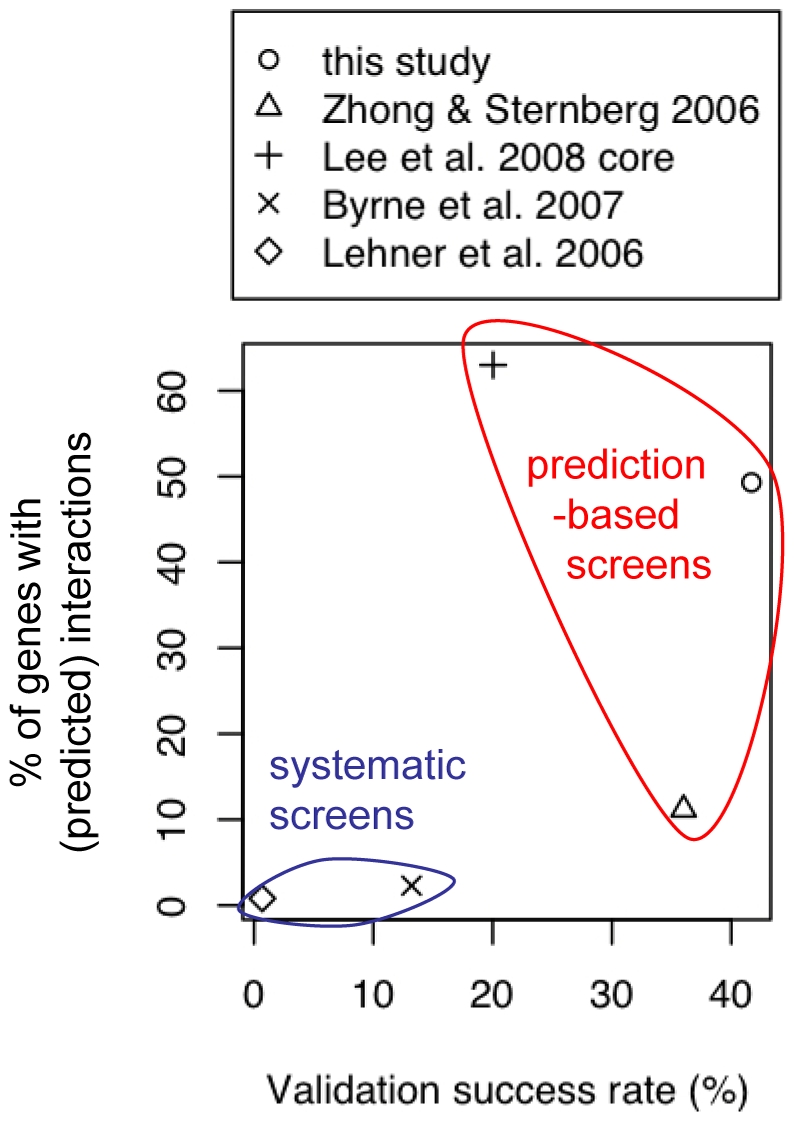
Comparison of large-scale genetic interaction studies in *C. elegans*. The studies are compared in terms of the percentage of genes with identified/predicted interactions and the success rate of experimental validation (i.e. the fraction of tested gene pairs that exhibit a genetic interaction). Systematic experimental screens test a limited number of gene pairs due to the labor-intensive experimental procedures. Moreover, these screens identify a small number of interactions relative to the number of tested gene pairs since genetic interactions appear to be rare. Prediction-based methods can assess all pairs of genes *in silico*, and consequently, the percentage of genes with predicted interactions tends to be larger than the percentage of genes with interactions identified by a systematic experimental screen. Moreover, predictions focus experimental efforts on gene pairs that are likely to exhibit a genetic interaction. Accordingly, the success rates of prediction-driven screens tend to be greater than the rates for systematic experimental screens. The success rate of our study shown here is conservative since it was computed based on the following definition: a gene pair exhibits an interaction only if the interaction is statistically significant according to all considered epistasis models (see Methods).

The nematode *Caenorhabditis elegans* is an ideal animal model for identifying genetic interactions due its genetic tractability. Furthermore, the high degree of conservation of molecular pathways related to human diseases has facilitated the dissection of physiopathological mechanisms of genetic disorders including Duchenne Muscular Dystrophy (DMD; OMIM: 310200), lysosomal storage disorders, obesity, diabetes and Huntington's disease [6]–[8]. Although the extent to which genetic interactions are conserved between *C. elegans* and human is unknown, previous studies encourage the use of *C. elegans* towards the identification of therapeutic targets for human diseases. For example, a genome-wide RNAi suppressor screen in a *C. elegans* model of type 2 diabetes, i.e. a strain with a loss-of-function mutation in the *C. elegans* insulin-like growth factor receptor *daf-2*, led to the identification of a kinase that exhibits antagonistic activity towards *daf-2*. Interestingly, mice with the kinase knocked-out appeared to be protected against diabetes, suggesting that the antagonistic interaction identified in *C. elegans* led to the identification of a potential therapeutic target for a human disease [8]. The application of systematic screens for other diseases hinges on the development of high-throughput techniques enabling the quantification of relevant phenotypes. However, the development of such quantitative techniques is in general time-consuming and may be extremely challenging. An alternative approach involves the *in silico* prediction of genetic interactions [9]–[11]. Interestingly, the rate at which genetic interactions are identified with prediction-driven screens appears to be significantly greater than the rate for systematic experimental screens (Figure 1). This suggests that *in silico* prediction represents an efficient approach to identifying genetic interactions.

All existing *in silico* approaches for predicting genetic interactions use several types of data including gene expression measurements and protein-protein (PP) interactions.

Lee and colleagues developed a method for predicting whether two given genes have a shared function [9]. The method is based on the weighted integration of gene pair data and was trained with pairs of genes that share functional annotations as positive learning examples. The predictions can be used in turn to infer genetic interactions, since pairs of genes that share function tend to exhibit synergistic interactions. Moreover, known antagonists of a given gene can be used as so-called seeds to search for other antagonists of the gene; specifically, genes predicted to share function with the seeds are inferred to be antagonists as well. However, many genes that share a function do not synergistically interact with each other nor do they antagonize the same gene(s), and therefore the accuracy of this approach for predicting genetic interactions may be limited (see the validation success rate in Figure 1).

Zhong and Sternberg developed a method to directly predict genetic interactions [10]. This method is also based on the weighted integration of gene pair data, but was trained with known genetic and PP interactions as positive learning examples. However, the method predicts a set of genetic interactions that involves only a small portion of all *C. elegans* genes (∼8% of the genome, see Figure 1). This may be due to the amount of data specific to a given gene pair that is required to make a prediction, since such data is scarce for many gene pairs.

Chipman and Singh developed an approach for predicting synergistic interactions only [11]. This approach uses information gained from the contexts of genes in a biological network that integrates several types of data (e.g. an edge exists between two genes if they encode proteins that exhibit a PP interaction), specifically by using the proximity between genes in the network. While this approach appears extremely powerful based on the *in silico* validation results, it remains to be determined how well this approach performs according to experimental validation.

**Figure 2:**
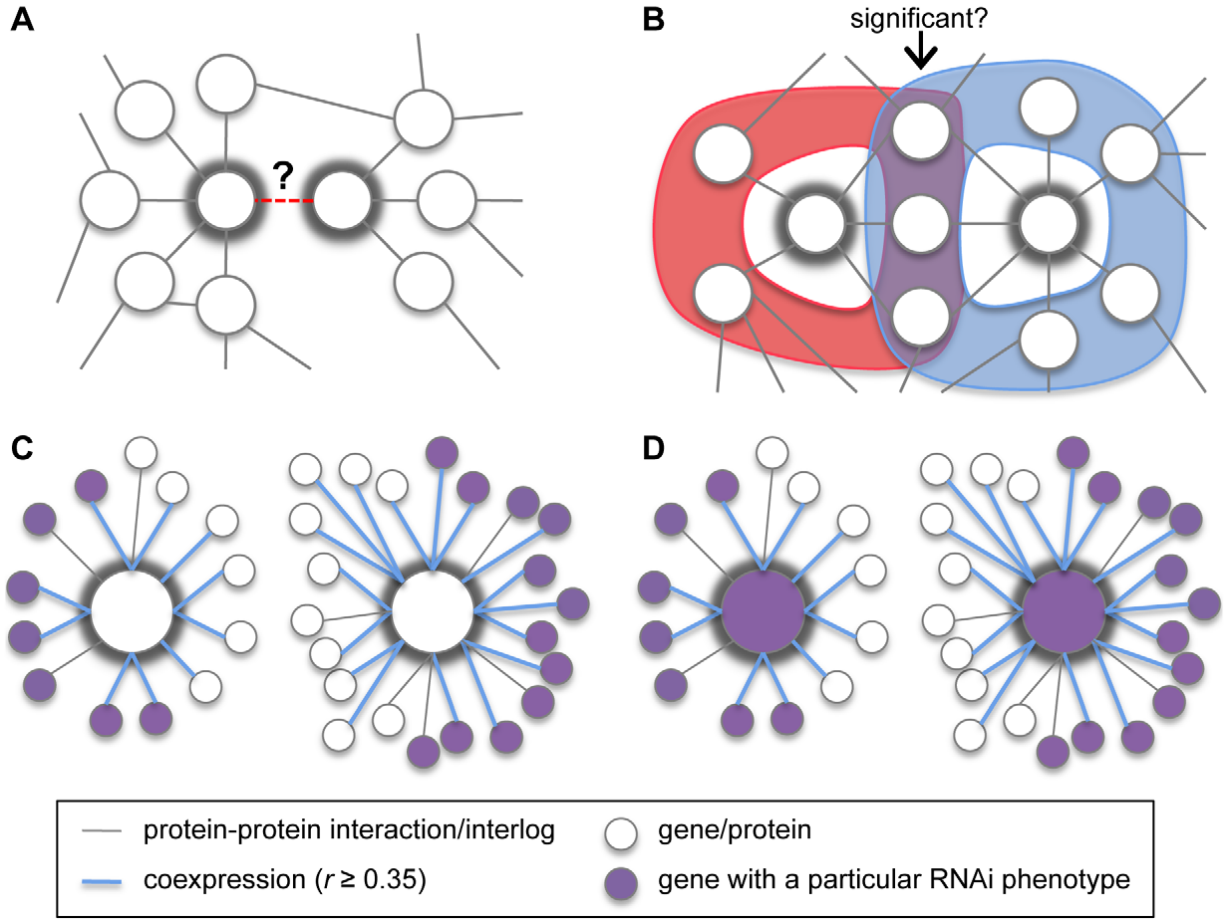
Gene pair attributes used to predict genetic interactions. The two genes/proteins of interest are highlighted with thick grey rings. (A) *I*, the presence or absence of a protein-protein (PP) interaction between the proteins encoded by the genes of interest, or their orthologues. (B) *CI*, a measure of the significance of the overlap between the PP interaction neighborhoods of the proteins encoded by the genes of interest (i.e. overlap of the red and blue regions). The PP interaction neighborhood of a given protein is the set of all of proteins that exhibit a PP interaction with the given protein (according to the multi-species PP interaction network). (C) *N*, an indicator for whether the neighborhoods of the genes of interest are enriched with the same phenotype. Here we define the neighborhood of a given gene as the set of genes that show significant coexpression (*P*≤0.05, see Methods) with the given gene and/or encode proteins that exhibit a PP interaction with the product of the given gene (according to the multi-species PP interaction network). Both neighborhoods shown here are enriched with a particular phenotype. (D) *NPh*, an indicator like *N* with the additional requirement that the genes of interest themselves must also exhibit the phenotype enriched in their neighborhoods.

Since all experimentally validated approaches for predicting genetic interactions currently suffer from limited accuracy or predict genetic interaction sets with limited genome coverage, we developed a novel *in silico* approach that uses statistical analyses of gene/protein neighborhoods in biological networks (Figure 2). Unlike previous approaches, the prediction of a genetic interaction between two given genes is aided by analyses that detect common features of the neighborhoods of the genes, or their encoded proteins (e.g. common PP interactors of the proteins). Furthermore, our approach does not require ‘seeds’ for every gene of interest to predict novel antagonistic interactions, unlike the Lee *et al.* approach [9], and while our approach appears comparable to the Zhong and Sternberg approach [10] in terms of specificity, our set of predicted genetic interactions has greater genome coverage (Figure 1).

The overall aim of this study was to identify genetic interactions in *C. elegans* that warrant further study in mammals towards the identification of promising therapeutic targets for genetic diseases. We thus used our approach to identify genetic interactors of the Rab-specific guanine-nucleotide dissociation inhibitor *gdi-1* (WormBase: WBGene00001558) which shares 80% protein sequence identity with *GDI1* (Ensembl: ENSG00000203879; Blast E-value: 2.10×10^−158^), a gene associated with non-syndromic forms of mental retardation in human (OMIM: 300104) [5]. *GDI1* encodes GDIα, a major regulator of Rab GTPase activity during endocytosis and exocytosis [5], [12]. This protein is thus a critical regulator of cell signaling events. The validation of predicted genetic interactions of *gdi-1* identified several antagonists. If these genetic interactions are conserved in the relevant human disease system, they would suggest therapeutic targets for *GDI1*-associated cognitive disorders. In addition, our results suggest the conservation of a subset of genetic interactions across different cellular systems in *C. elegans*, and the involvement of *GDI1* in human myopathies resulting from mutations in components of the Dystrophin Glycoprotein Complex (DGC).

## Results

### The predictor of genetic interactions in *C. elegans*

We developed a predictor of genetic interactions using a learning set that contains positive and negative examples of interactions from the literature (see Table S1 for the manually-curated interactions) and gene pairs randomly selected from the *C. elegans* genome, respectively (see Methods). Since it is estimated that the vast majority of gene pairs do not genetically interact [13], a set of randomly selected gene pairs is expected to be enriched with true negative examples.

Our predictor uses gene expression measurements, RNAi knockdown phenotype observations and PP interactions from multiple species to measure the likelihood of a genetic interaction. The gene expression measurements were obtained from DNA microarray results [14], and the phenotype observations were obtained from genome-wide RNAi experiment results (see Methods). A multi-species PP interaction network was constructed with *C. elegans*, *Drosophila melanogaster*, *Homo sapiens* and *Saccharomyces cerevisiae* PP interactions identified by yeast two-hybrid (obtained from BioGRID and [15], [16]). PP interactions from species other than *C. elegans* were incorporated using InParanoid orthology maps [17]. For any two given genes, we considered a measure of their coexpression (*Exp*), a measure of their phenotype similarity (*Ph*) and an indicator of a PP interaction between their encoded proteins or orthologues (*I*) as gene pair attributes that might help determine whether the given genes genetically interact. Variants of the *Exp*, *Ph* and *I* attributes have been used by existing predictors of genetic interactions [9], [10].

Importantly, our approach is the first to use particular features of biological networks in order to improve the accuracy of the prediction of genetic interactions. For example, considering the multi-species PP interaction network, we define the neighborhood of a protein as the set of proteins that exhibit a PP interaction with it (possibly via orthology). Although two given proteins may not be known to exhibit a PP interaction, their neighborhoods may contain a surprising number of common PP interactors (Figure 2B). We defined a gene pair attribute based on the encoded proteins of the two genes of interest, measuring the significance of their number of common PP interactors (*CI*). The set of gene pairs that encode proteins with significantly many common PP interactors (*CI*≤0.05) is enriched with gene pairs that are known to genetically interact (*P* = 6.67×10^−39^, hypergeometric test).

We also investigated whether a biological network that integrates observations of phenotype similarity, coexpression and PP interaction can improve the prediction of genetic interactions. We defined a novel biological network called the PhEP network, where nodes represent genes and the genes are labeled with their RNAi knockdown phenotypes. Two genes are connected by an edge if they are significantly coexpressed and/or if they encode or have orthologous proteins that exhibit a PP interaction (see Methods). Although a phenotype observation may be absent for the gene itself, such observations may be available for several of its neighbors (i.e. the genes connected to the gene of interest by one edge) in the PhEP network. We therefore defined a gene pair attribute indicating the enrichment of genes associated with some phenotype in the neighborhoods of both genes of interest, in the PhEP network (*N*, see Figure 2C). We demonstrated that the set of gene pairs with such neighborhood characteristics is enriched with gene pairs that are known to genetically interact (*P* = 1.20×10^−245^, hypergeometric test). By the same line of reasoning, we defined a variant of this gene pair attribute, *NPh*, indicating that the two genes of interest are annotated with the phenotype that is enriched in both of their neighborhoods in the PhEP network (see Figure 2D). Again, the set of gene pairs with such neighborhood characteristics is enriched with gene pairs that genetically interact (*P* = 6.26×10^−113^, hypergeometric test).

Taken together, we showed that our network-based attributes (*CI*, *N* and *NPh*) of gene pairs are significantly associated with genetic interactions, suggesting that these attributes may facilitate the accurate prediction of genetic interactions.

Ultimately, the *Exp*, *Ph*, *I*, *CI*, *N*, and *NPh* attribute values of a given gene pair are integrated by a logistic regression model that outputs a prediction score between 0 and 1 representing the likelihood of a genetic interaction between the two genes (see Methods).

We performed leave-one-out cross-validation to evaluate the predictor at different score thresholds (Figure S1). We determined that a conservative threshold of 0.975 induces error rates comparable to those achieved by the Zhong and Sternberg (ZS) genetic interaction predictor (Table S2). However, this threshold also induces a set of predicted genetic interactions that is 98% novel when compared to the prediction sets of previous studies [9], [10], and roughly three-fold more genes are present in our set compared to the ZS set. Thus, under conditions where our predictor and the ZS predictor have comparable accuracy estimates, our set of predicted interactions exhibits greater genome coverage. We chose 0.85 as our definitive threshold since it yields an estimated false positive rate (Table S2) close to the expected rate of finding a genetic interaction at random (0.5%) [13], coinciding with our negative learning set of random gene pairs. At this threshold, the estimated true positive rate is 10.8% (Table S2). Although our predictor misses many true positive interactions, over 800K genetic interactions are predicted and again, 98% of them are novel (Figure S2A). In particular, our predictor proposes more interactions per gene on average compared to the ZS predictor (Figure S2B). In addition, roughly four-fold more genes are present in our set of predicted interactions compared to the ZS set (Figure S2B). Thus, when the predictor has an estimated false positive rate that is appropriately low, the corresponding set of predicted interactions also exhibits a large increase in genome coverage compared to the ZS set. Genome-wide genetic interactions predicted by our method are available online (http://www.mcb.mcgill.ca/~anna/gInterWorm/search.php).

**Figure 3:**
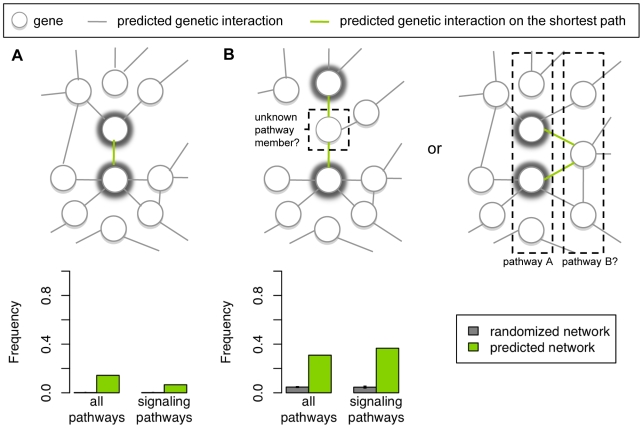
Assessment of the biological relevance of the predicted genetic interaction network with pathway annotations. Here we show scenarios where a pair of genes annotated to the same pathway (A) is directly connected or (B) shares ≥1 neighbor in a genetic interaction network, where the gene pair of interest is highlighted with thick grey rings. In (A), the genes exhibit a within-pathway genetic interaction based on the given set of pathway annotations. In (B), the genes belonging to the same pathway (e.g. pathway A) both interact with a gene that may either be an unknown member of the same pathway (within pathway interaction), or may belong to a different pathway (e.g. pathway B, between-pathway interactions). Below, the frequencies at which each scenario occurs in the predicted network and in randomized networks are shown with respect to all pathways and to signaling pathways only (see Methods). The “all pathways” and signaling pathway annotations were derived from human and *C. elegans* experimental data respectively. For each set of pathway annotations, the median, first and third quartile frequencies of each scenario were computed across *N* = 100K randomized networks; the bar length depicts the median and the error bars depict the first and third quartiles. Both scenarios occur more frequently than what is expected by chance, for both sets of pathway annotations.

The biological relevance of the predicted genetic interaction network was assessed *in silico* using pathway annotations ([18] and Table S3). Previous studies show that genetic interactions occur within and between pathways, although between-pathway interactions are more prevalent amongst interactions identified in large-scale studies [19]–[21]. Therefore, we investigated the connectivity of pairs of genes annotated to the same pathway, in the predicted network (see Methods). We found that a significant fraction of these pathway gene pairs are directly connected (*P* = 10^−5^, Figure 3A), indicating predicted interactions within pathways. We also found that a significant fraction of pathway gene pairs are connected through shared neighbors (*P* = 10^−5^, Figure 3B), and in most cases, at least one of the shared neighbors is not in the same pathway as the pair (98% and 99% of the cases for all and just signaling pathways, respectively). These cases indicate predicted interactions that likely occur between pathways, or within a pathway if the shared neighbor is an unknown member of the pathway of the pair. Interestingly, we predict significantly many genetic interactions within and between pathways mapped from human to *C. elegans*, as we do for pathways derived directly from *C. elegans* (compare “all pathways” to “signaling pathways” in Figure 3). Taken together, the connectivity of pathway genes in the predicted network is consistent with connectivity observations based on genetic interactions identified experimentally [19]–[21], even for pathway genes mapped from human, and thus supports the validity of our predictor.

### The set of predicted genetic interactions exhibits improved coverage of genes conserved between human and *C. elegans*

We investigated whether more genes conserved between human and *C. elegans* are present in our set of predicted genetic interactions when compared to other prediction sets. When all prediction sets are restricted to genes with human orthologues (see Text S1), it is still true that a large fraction of our set is novel (Figure S2A). We thus examined the level of characterization of human genes with *C. elegans* orthologues present in prediction sets. Our analysis shows that *in silico* methods tend to predict genetic interactions involving well-characterized genes more often than poorly-characterized genes (Figure S2C). All human genes with *C. elegans* orthologues only present in the ZS prediction set have a high level of characterization (gene characterization index >5 [22]). Interestingly, 25% of human genes with *C. elegans* orthologues only present in our prediction set do not have a high level of characterization. Taken together, our approach predicts a large number of novel genetic interactions for genes conserved between *C. elegans* and human, and also predicts interactions for genes orthologous to poorly-characterized human genes that have no predicted interactions by other approaches.

In order to better understand why our method predicts genetic interactions that are mostly novel, we investigated the genes with human orthologues associated with mental retardation and synaptic plasticity (MRSP) that we curated from the literature (Table S4). Over two-fold more MRSP genes are present in our set of predicted genetic interactions compared to the ZS set (89% and 40% of the genes, respectively). In examining the MRSP genes that are present in our set only, we found that these genes are generally associated with more information with our approach than with the ZS approach (see Figure S3A and the Methods). In particular, the additional information comes from our novel network-based attributes (e.g. the *CI* and *N* attributes). The values of these attributes are computable for nearly all MRSP genes, but the values of most ZS attributes are computable only for a smaller subset of the genes (Figure S3B). These results suggest that the network-based attributes facilitate the prediction of novel interactions.

A large number of human genes associated with disease are conserved in *C. elegans* [23]. For example, *GDI1*, a human gene associated with mental retardation [5], has high sequence similarity (Blast E-value: 2.10×10^−158^) to *gdi-1*, a *C. elegans* gene that has yet to be functionally characterized. Since *GDI1* is involved in neurotransmission and has been associated with cognitive deficiency in human [5], [12], it is functionally related to our set of human MRSP genes (Table S4). We thus investigated whether the relationship between *GDI1* and MRSP genes is conserved between human and *C. elegans*. Interestingly, our method predicts that *gdi-1* genetically interacts more frequently with MRSP genes than with other genes (*P* = 1.1×10^−5^, two proportion test), and it also shares genetic interaction partners more frequently with MRSP genes than with other genes (*P* = 1.1×10^−50^, two proportion test). These results provide statistically significant evidence that the interactions between *GDI1* and its potential neurological partners are conserved in *C. elegans*.

### Validation of predicted genetic interactors of *gdi-1*

**Figure 4:**
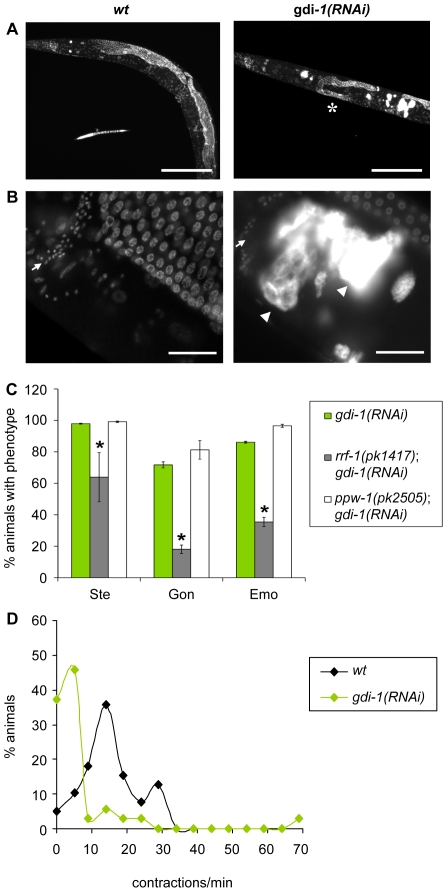
Phenotypical characterization of *gdi-1(RNAi)*-treated animals. (A,B) DAPI staining of *egfp(RNAi)*- (labeled *wt*) and *gdi-1(RNAi)*-treated wild-type animals. (A) Gonad morphogenesis defects (Gon) characterized by short gonads (*) are observed in *gdi-1(RNAi)*-treated animals. Scale bar, 200 µm. (B) Accumulation of Endomitotic oocytes (Emo, arrowheads) in the proximal gonad of *gdi-1(RNAi)*-treated animals. Arrows indicate the spermathecae. Scale bar, 25 µm. (C) Sterility (Ste), Gon and Emo phenotypes were measured in wild-type, *rrf-1(pk1417)* and *ppw-1(pk2505)* animals submitted to *gdi-1(RNAi)* (*N* = 3). The mean expressivity/penetrance of each phenotype is shown with error bars representing ± one standard error. A (*) indicates a statistically significant reduction of the phenotypes (*P*≤0.05, Student's *t*-test) compared to wild-type animals treated with *gdi-1(RNAi)*. (*D*) Distributions of the sheath cell contraction frequency for *egfp(RNAi)*- (labeled *wt*) and *gdi-1(RNAi)*-treated animals.

We identified phenotypes that result from treating *C. elegans* animals with *gdi-1(RNAi*). These phenotypes include sterility (Ste, Figure 4C), a gonad morphogenesis defect characterized by a shortening of gonads (Gon, Figure 4A,C), an ovulation defect characterized by an accumulation of endomitotic oocytes (Emo, Figure 4B,C), and a severe reduction of sheath cell contraction (Figure 4D). We showed that *gdi-1* controls ovulation and gonad morphogenesis processes by modulating somatic gonad cell functions. That is, *rrf-1(pk1417)* (WormBase: WBGene00004508) animals, which are resistant to RNAi in somatic cells, expressed significantly reduced levels of the phenotypes when subjected to *gdi-1(RNAi)* compared to wild-type and mutant animals resistant to RNAi in germinal cells (see *gdi-1(RNAi)* and *ppw-1(pk2505)*(WormBase: WBGene00004508); *gdi-1(RNAi)* respectively in Figure 4C). These results suggest that *gdi-1* is a critical regulator of signaling pathways controlling reproductive functions in *C. elegans*.

**Figure 5:**
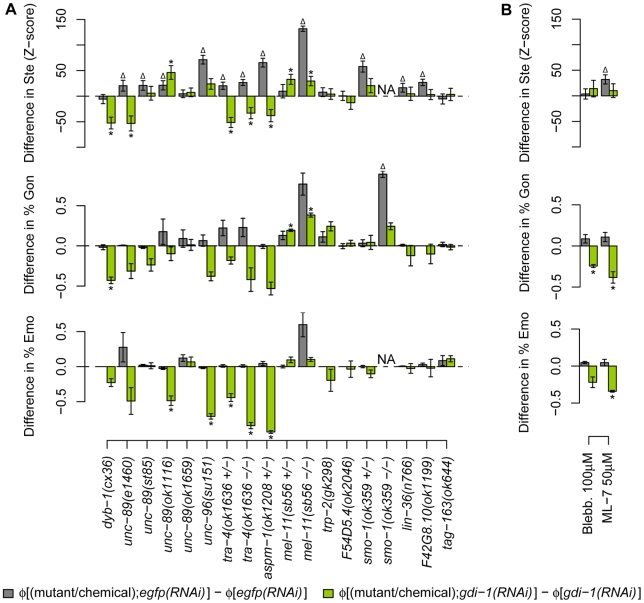
Validation of a subset of genetic interactions predicted for *gdi-1*. Ste, Gon and Emo phenotypes were measured in animals submitted to RNAi against *egfp* (grey) or *gdi-1* (green). The mean difference in the expressivity/penetrance of each phenotype in perturbed (mutant or chemically treated) versus wild-type (wt) animals (denoted φ[*x*]−φ[*y*], for animals of type *x* and *y*) is shown with error bars representing ± one standard error, *N*≥3. For Ste, the *Z*-score of the difference in expressivity is plotted (see Text S1). (Δ) and (*) indicate statistically significantly differences for animals treated with *egfp(RNAi)* and *gdi-1(RNAi)*, respectively (*P*≤0.05, see Methods). NA: not available. (A) Differences in phenotype expressivity induced by mutations in genes predicted to interact with *gdi-1*. (B) Differences in phenotype expressivity induced by chemical treatment. Blebbistatin (Blebb.) is a myosin ATPase inhibitor and ML-7 is a specific inhibitor of myosin light-chain kinase.

**Figure 6:**
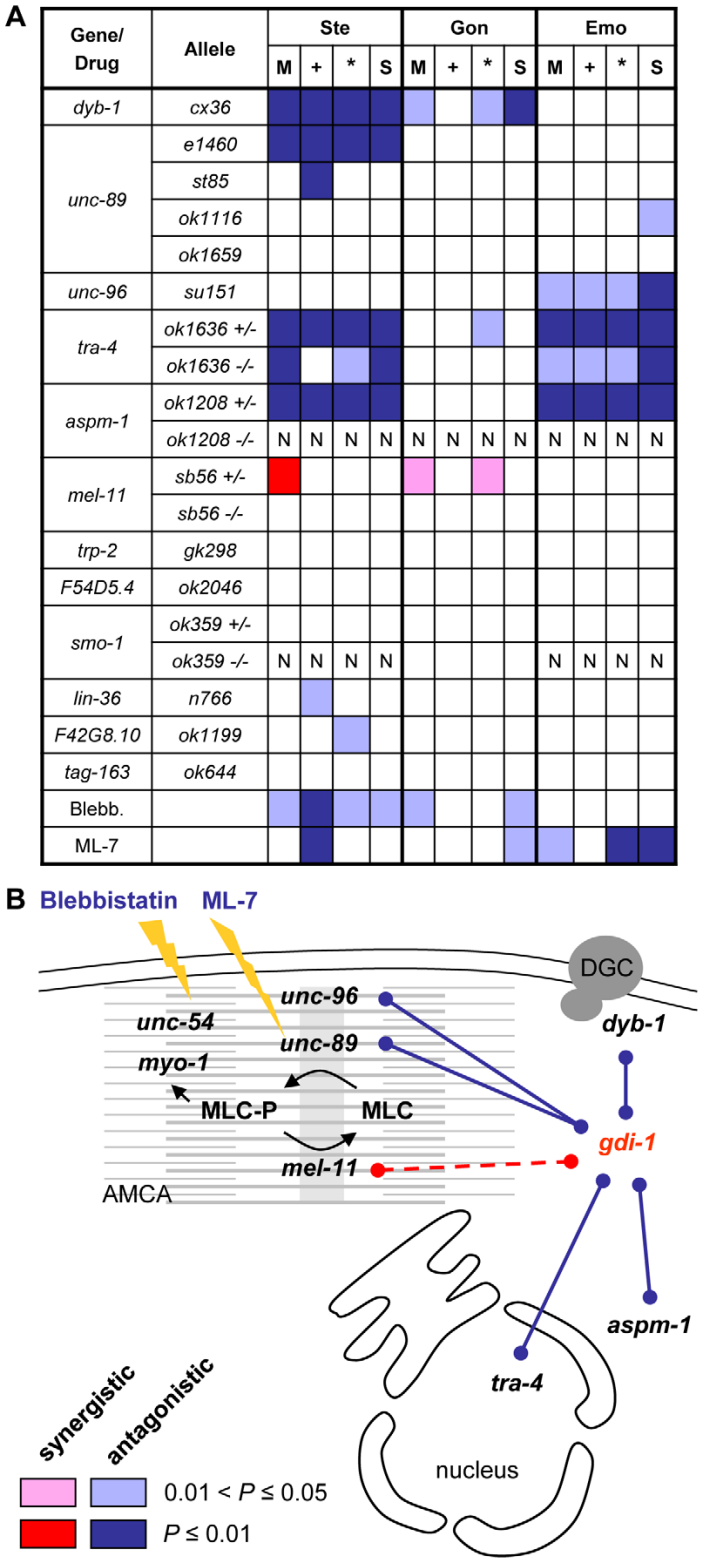
Epistasis between *gdi-1* and its predicted genetic interactors and chemical suppressors. (A) The minimum (M), additive (+) and multiplicative (*) statistical models of epistasis were used in the analysis. A statistical test for the specific suppression (S) of *gdi-1(RNAi)*-induced defects was also used (see Methods for details). Significant synergistic and antagonistic interactions are illustrated with shades of red and blue, respectively (*P*≤0.05). Darker shades indicate significant interactions with *P*≤0.01. The absence of a statistically significant interaction is indicated by a white entry. NA: not available. (B) Schematic representation of *gdi-1* interactors. Blue lines represent antagonistic interactions with *gdi-1*. The dashed red line indicates phenocopy between *mel-11* and *gdi-1*. *unc-96* (paramyosin-binding protein), *unc-89* [myosin light chain (MLC)-kinase], and *mel-11* (MLC-phosphatase) are regulators of the actin-myosin contractile apparatus (AMCA, represented in grey) [26], [72]. *unc-54* and *myo-1* are type II myosin heavy chains. *tra-4* encodes a *PLZF*-like transcription factor [28]. *aspm-1* (orthologue of mammalian *ASPM*) and *dyb-1* (orthologue of a component of the dystrophin glycoprotein complex, DGC) have been associated with mitotic spindle assembly and DGC function in human, respectively [32]. ML-7 and blebbistatin (Blebb.) are specific inhibitors of MLC-kinase and myosin II ATPase activities, respectively.

To experimentally validate our predictions, we examined 18 strains containing mutations in 12 genes predicted to genetically interact with *gdi-1*. Ste, Emo and Gon phenotypes were measured for mutant and wild-type animals submitted to RNAi against *gdi-1* or the negative control, *egfp* (Figure 5A). Epistasis analyses of these measurements were performed using three commonly used statistical models [24], [25] to identify significant genetic interactions (see Tables S5 and S6 for the estimated epistasis coefficients and *P* values, respectively). We also applied a statistical test that measures the suppression of *gdi-1(RNAi)*-induced phenotypes (see Methods). Our results show only partial agreement between the different models of epistasis (Figure 6A). The most stringent requirement (i.e. significant interaction by all applicable tests, *P*≤0.05) resulted in a validation success rate of 42%, while more permissive analysis (i.e. significant interaction by at least one test) increased the success rate to 67%. This represents an 84- or 134-fold improvement over the expected success rate from random genetic screening. Although validation success rates depend on the selected bait gene(s) (e.g. *gdi-1* in our study), our success rates surpass those reported for existing methods [9], [10] (Figure 1A), thus suggesting that our method represents an important improvement in predictive accuracy.

Of the 12 putative genetic interactions tested, five were successfully validated according to all statistical tests used to analyse our results. All five interactions are antagonistic, and the genes that antagonize *gdi-1* are the following: *unc-96* (WormBase: WBGene00006825), encoding a paramyosin-binding protein [26]; *unc-89* (WormBase: WBGene00006820), encoding a titin-like myosin light chain (MLC)-specific kinase [27]; *tra-4* (WormBase: WBGene00018740), a close orthologue of the human proto-oncoprotein and transcriptional repressor *PLZF* (Ensembl: ENSG00000109906) [28]; *aspm-1* (WormBase: WBGene00008107), the closest orthologue of the mammalian *ASPM* (Ensembl: ENSG00000066279), a gene associated with mitotic spindle assembly and microcephaly [29], [30]; and *dyb-1* (WormBase: WBGene00001115), the closest orthologue of dystrobrevin (Ensembl: ENSG00000134769), a component of the DGC in human [31].

Notably, we showed genetic interactions between *gdi-1* and regulators of the actin-myosin contractile apparatus. Indeed, *gdi-1*-associated phenotypes were reduced by a mutation in the MLC-specific kinase (MLCK) *unc-89*, while *gdi-1(RNAi)* phenocopies a mutation in the MLC-specific phosphatase *mel-11* (WormBase: WBGene00003196; Figure 5A). This suggests that *gdi-1* antagonizes MLC phosphorylation and consequently, contraction through the actin-myosin apparatus during gonad morphogenesis. Consistent with these results, *gdi-1*-associated phenotypes were reduced by a chemical inhibitor of MLCK (ML-7) and a chemical inhibitor of myosin II ATPase activity (blebbistatin) (Figures 5B and 6).

**Figure 7:**
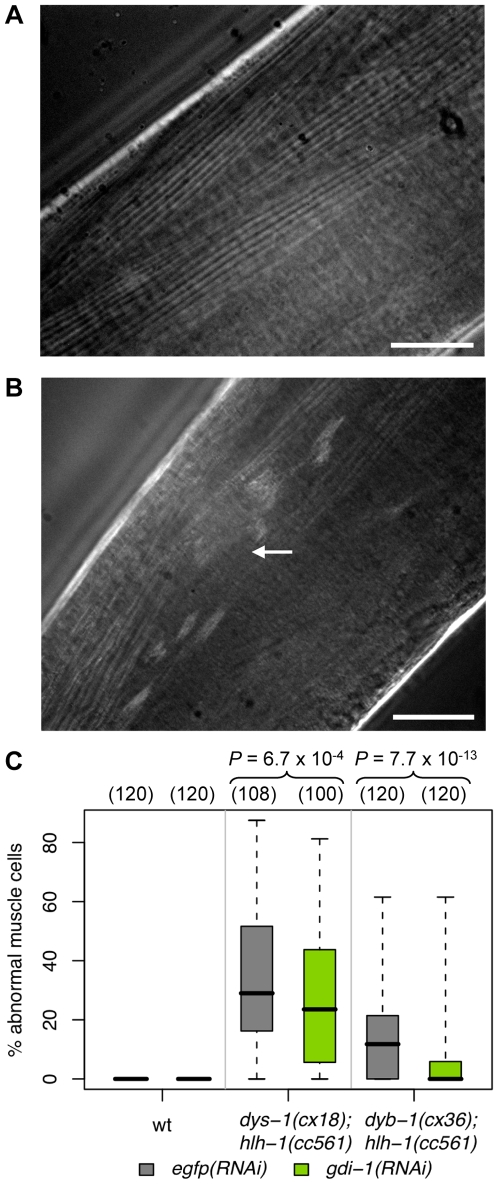
*gdi-1* suppresses *dys-1*- and *dyb-1*-associated muscle degeneration. Body-wall muscle fibers observed using polarized light microscopy in (A) wild-type and (B) *dys-1(cx18);hlh-1(cc561)* animals. The arrow indicates an abnormal/degenerated muscle cell. Scale bar, 200 µm. (C) Muscle degeneration was assessed in wild-type (wt), *dys-1(cx18);hlh-1(cc561)* and *dyb-1(cx36);hlh-1(cc561)* animals submitted to RNAi against *egfp* (grey) or *gdi-1* (green). The percentage of abnormal muscle cells in a methanol fixed animal, estimated with polarized light microscopy, was used to quantify muscle degeneration in the animal. Boxplots of these percentages are shown. The total number of animals assessed across three independent experiments is shown above each boxplot in parentheses. The percentage of abnormal muscle cells is significantly reduced in *gdi-1(RNAi)*-treated versus *egfp(RNAi)*-treated mutant animals as indicated by the *P* values shown at the top (see Methods).

We also identified a genetic interaction between *gdi-1* and *dyb-1* that affects gonad morphogenesis (Figures 5A and 6). The latter gene is a close orthologue of dystrobrevin, a component of the DGC that when altered leads to myopathies [32]. Moreover, dystrobrevin is a functional partner of dystrophin (Ensembl ENSG00000198947), a protein that is associated with DMD and mild cognitive deficiencies in human [32]. *C. elegans* is a model organism used to dissect the molecular mechanism of myopathy associated with mutations in the DGC components *dyb-1* and *dys-1* (WormBase: WBGene00001131, the orthologue of dystrophin) [33]. As shown previously, mutations in *dyb-1* and *dys-1* produce a progressive myopathy when combined with a weak allele of *hlh-1* (WormBase: WBGene00001948; compare panels A and B of Figure 7) [34], [35]. We showed that *gdi-1(RNAi)* treatment significantly reduces muscle degeneration in *dyb-1(cx36);hlh-1(cc561)* and *dys-1(cx18);hlh-1(cc561)* mutants (Figure 7C). Therefore, we demonstrated that the antagonism between *gdi-1* and *dyb-1* is conserved in different cellular systems in *C. elegans*.

Taken together, our experimental results identified genes that antagonize *gdi-1* activity during gonad morphogenesis, ovulation (Figure 6B), and muscle degeneration.

## Discussion

We present a prediction-based approach to identifying genetic interactions in *C. elegans*. The approach predicts many novel interactions, including interactions for poorly-characterized genes. Our validation results for *gdi-1* suggest that our predictions identify true interactions with a success rate far beyond random genetic screening (i.e. at least 84-fold greater than the rate of identifying true interactions by chance), and that our approach has improved accuracy compared to previous approaches. Moreover, we identified five genes with antagonistic activities towards *gdi-1* activity during gonad morphogenesis and/or ovulation, including genes associated with phosphorylated MLCs and *dyb-1*. Interestingly, we also showed that the antagonism between *gdi-1* and *dyb-1* influences muscle cell morphology.

Our predictor integrates novel attributes based on network analysis. We showed that each network-based attribute identifies gene pairs that are enriched for true genetic interactions. The common interactors (*CI*) attribute is based on the common PP interactors of the proteins encoded by the two genes of interest, in a multi-species PP interaction network. Two given proteins that have surprisingly many common PP interactors may be members of the same complex, thereby increasing the likelihood that their encoding genes genetically interact, since members of the same complex tend to genetically interact [13], [36]. Moreover, the *N* and *NPh* attributes are based on shared phenotypes in a so-called PhEP network constructed with RNAi knockdown phenotype, gene expression and PP interaction data. When a specific phenotype is associated with surprisingly many neighbors of a given gene in the PhEP network, it may follow that the gene modulates this phenotype. Thus, if the neighborhoods of two given genes are characterized by the same phenotype(s), the genes may modulate the same phenotype(s), thereby increasing the likelihood that they genetically interact. Furthermore, the network-based attributes provide additional information for less-studied genes, such as genes that may not have been assayed individually (e.g. for phenotype observations) or with other genes systematically (e.g. for PP interactions). For example, no phenotypes have been observed for *unc-89* and it has not been tested for a PP interaction with *gdi-1*. However, the *CI* and *N* attributes support a genetic interaction between *unc-89* and *gdi-1*, which we confirmed experimentally. This suggests that the network-based attributes facilitate the accurate prediction of genetic interactions.

Our analyses suggest that *in silico* approaches tend to predict genetic interactions involving well-characterized genes more often than poorly-characterized genes. The Zhong and Sternberg (ZS) approach explicitly restricts the predictions to genes that satisfy a minimum information requirement (i.e. a gene must be associated with information from at least one attribute that is not the *C. elegans* gene expression attribute) [10]. Only ∼50% of all genes satisfy the requirement. As a result, only ∼25% of all genes pairs are tested *in silico*. In our approach, we do not impose a minimum information requirement. Moreover, we gained information for ∼80% of all gene pairs by integrating our network-based attributes. These features of our approach may be responsible for the large number of novel predicted genetic interactions.

All of the experimentally validated interactions are antagonistic. This suggests that our learning set contains a strong signal for antagonistic interactions and that our approach captures this signal. If this is the case, our approach may be advantageous for predicting antagonistic interactions. Consequently, our approach may also be advantageous for proposing antagonisms that warrant further study in mammals towards the identification of therapeutic targets for monogenic disorders.

Because of its involvement in vesicular trafficking in mammals, *GDI1* may be a critical regulator of several signaling pathways controlling functions such as synaptic plasticity, learning and memory acquisition [5], [37], [38]. Interestingly, the signaling pathways involving ephrins, integrins and inositol-triphosphate that control gonad morphogenesis and ovulation in *C. elegans* are highly similar to the pathways controlling synaptic plasticity in human [39]–[44]. Supporting this observation, the anti-epileptic drug valproate, which targets components of these signaling pathways in human, has been shown to cause severe alteration of sheath cell contraction and ovulation processes in *C. elegans* [45]. Moreover, our data suggest that *gdi-1*, like valproate [45], controls ovulation processes by modulating somatic gonad cell functions. As documented by the Gilbert and Bolker study [46], a conserved signaling pathway can control different cellular processes in different organisms; for example, ovulation in *C. elegans* versus synaptic plasticity in human. However, a signaling pathway that is conserved across different cellular systems and species may have also acquired some context-specific signaling components. We therefore do not expect all signaling pathway observations in one context to apply to another context. However, a number of genes identified as genetic interactors of *gdi-1*, using Ste, Gon and Emo as phenotypical readouts in nematodes, have high sequence similarity to genes with neurological functions in human. These observations support the search for genetic interactors of *gdi-1* with a role in controlling gonad morphogenesis and ovulation in *C. elegans* to suggest likely genetic interactors of *GDI1* controlling cognitive abilities in human. Nevertheless, this strategy for identifying genetic interactions relevant to cognition requires extensive validation in higher organisms such as mouse.

One of the genetic interactions that we uncovered is between *gdi-1* and *aspm-1*. In both *C. elegans* and mammals, *aspm-1* controls mitotic spindle positioning and consequently, the ratio of symmetric and asymmetric cell divisions [29], [47]. While control of asymmetric division of somatic gonadal precursor cells (SGPs) is required for the proper morphogenesis of gonads in *C. elegans* [48], it is still unknown whether the modulation of asymmetric division in these cells is at the origin of the interaction between *gdi-1* and *aspm-1*. *aspm-1* is an orthologue of *ASPM*, a gene involved with brain development in human. In particular, *ASPM* is involved in the control of neuronal progenitor proliferation and is associated with microcephaly [47]. Since *GDI1* is expressed in both proliferative and differentiated neurons during brain development [5], it would be interesting to test whether *GDI1* genetically interacts with *ASPM* in mammalian brains and consequently, test if the simultaneous perturbation of both genes would result in a reduction of cognitive disabilities associated with mutations in either *ASPM* or *GDI1* alone.

The molecular origin of the genetic interaction observed between *gdi-1* and *tra-4* is also unknown. The transcriptional repressor *tra-4* was shown to promote female development by repressing male-specific genes in *C. elegans* [28]. This gene was also characterized as a SynMuvB gene because it was shown to negatively regulate *let-60* (WormBase: WBGene00002335)/Ras-mediated vulval development in nematodes [28]. Interestingly, several SynMuvB genes have been shown to control somatic gonad development [49], [50]. Further studies will be required to assess the function of *tra-4* during somatic gonad development and its potential interaction with SynMuvB genes in the cellular context of that process.

We also showed genetic interactions between *gdi-1* and regulators of the actin-myosin contractile apparatus. Indeed, *gdi-1*-associated phenotypes were reduced by mutations in the MLCK *unc-89* and its functional partner *unc-96* [26]. Interestingly, *unc-96* is required for the proper distribution of *unc-89* at the M-line in body-wall muscle sarcomeres [26]. Our data suggest a partnership between *unc-96* and *unc-89* that promotes the contraction of the actin-myosin contractile apparatus in gonad somatic cells, in a pathway antagonistic to *gdi-1*. This hypothesis is also supported by the significant reduction of *gdi-1(RNAi)*-induced phenotypes in animals treated with the MLCK and myosin II inhibitors ML-7 and blebbistatin, respectively.

Interestingly, MLC phosphorylation and myosin II function have been shown to control synaptogenesis, dendritic spine morphology and synaptic plasticity in mammals [51], [52]. Moreover, the inhibition of MLCK function in the lateral amygdala of the mouse brain has been shown to enhance auditory fear conditioning (i.e. learning and memory) and to facilitate synaptic plasticity [53]. Because mutating *GDI1* in mice has the opposite effect [54], it is of interest to assess whether phosphorylated MLC and *GDI1* have antagonistic functions in neurological mechanisms that enable learning and memory acquisition in mammals. If antagonism is present, inhibiting phosphorylated MLC is a potential therapeutic strategy to reduce the symptoms associated with *GDI1* mutations in human.

We also demonstrated that the activity of *gdi-1* is antagonistic with the activity of the dystrobrevin orthologue, *dyb-1*, during gonad morphogenesis in *C. elegans*. Dystroglycan is another component of the DGC and its orthologue *dgn-1* has been previously shown to control gonad morphogenesis in *C. elegans* [55]. Our data suggest that *dyb-1* also contributes to this developmental process. Interestingly, the antagonism between *dyb-1* and *gdi-1* is consistent with the likely antagonism in mammals where dystrobrevin acts as a regulator of cell signaling through the inhibition of receptors and membrane recycling [56], and *GDI1* potentially promotes these cycling events by regulating *RAB4* and *RAB5* [38]. We also showed that this antagonism is conserved in different cellular systems in *C. elegans* since *gdi-1(RNAi)* treatment significantly reduced muscle degeneration in both *dyb-1;hlh-1* and *dys-1;hlh-1* animals. Mechanisms of muscle degeneration resulting from functional alterations of dystrobrevin or dystrophin are still poorly understood in mammals [57]. While *C. elegans* is an animal model of choice to dissect the pathological mechanisms associated with myopathies [58], the antagonisms observed between the *GDI1*, dystrobrevin and dystrophin orthologues in *C. elegans* should be confirmed in DMD mammalian models (e.g. the mdx mouse) before considering *GDI1* as a promising therapeutic target for DMD. Furthermore, since DGC components and *GDI1* are expressed at the synapses of hippocampus neurons [34], [38] it would be extremely interesting to test whether perturbations of dystrobrevin function may reduce cognitive disabilities associated with mutations in *GDI1* in mammals.

In summary, we developed a bioinformatics tool to predict genetic interactions in *C. elegans* towards the identification of therapeutic targets to address monogenic disorders associated with disruptions in signaling homeostasis. Our tool uses network-based attributes and our validation suggests that it predicts interactions more comprehensively and with improved accuracy compared to other tools. In addition, we experimentally confirmed the interactions that were predicted between *gdi-1* and several genes involved in neurological functions in human. Notably, we established that perturbation of *aspm-1*, *tra-4*, *unc-89*, *unc-96*, or *dyb-1* reduces the signaling unbalance resulting from a reduction of *gdi-1* expression. We also showed that a reduction of *gdi-1* expression significantly reduces muscular dystrophy in nematode DMD models. Further studies using relevant mammalian models are required to assess whether *ASPM*, MLC phosphorylation machinery and dystrobrevin would be potent therapeutic targets for cognitive disabilities associated with mutations in *GDI1*. Similarly, further studies in mammalian models would be required to assess whether *GDI1* would be a potent therapeutic target for DMD. In conclusion, we have developed a valuable tool that facilitates the mapping of genetic interactions in *C. elegans*. Since the conservation of pathogenic mechanisms and genetic interactions between distant species is still under intense debate, experimental validation in mammals of genetic interactions identified in *C. elegans* is required to evaluate the potential of our method to significantly streamline the therapy development process for monogenic disorders that involve genes and signaling pathways conserved between human and *C. elegans*.

## Methods

The development and subsequent analysis of the genetic interaction predictor were completed in the R v2.6 statistical computing environment (http://www.r-project.org, [59]).

### Construction of the learning set

A learning set, comprised of a positive and a negative subset, was constructed for the training of the predictor of genetic interactions. The positive learning set consists of 1,522 genetic interactions identified by automated [60] or manual curation of the literature (see Table S1). The negative learning set should consist of pairs of non-interacting genes. Since the vast majority of gene pairs are believed not to genetically interact [13], we built our negative learning set from ∼14,000 randomly selected gene pairs from the set of all genes mapped to a genomic location (WormBase release WS180, http://www.wormbase.org/). The approximate 1∶10 ratio of positive to negative interactions was established to guarantee a learning set with a thorough sampling of all gene pair combinations (∼386 million in total).

### Datasets used to derive attributes

The gene expression data was obtained from [14]. We obtained all RNAi knockdown phenotype data in WormBase release WS141 and removed seven uninformative or redundant types, such as “wildtype”, “unclassified”, “not embryonic” and “complex phenotype.” Protein-protein (PP) interactions were obtained from all *C. elegans*, *Saccharomyces cervisiae*, *Drosophila melanogaster*, and *Homo sapiens* yeast two-hybrid datasets stored in BioGRID v2.0.37 (http://www.thebiogrid.org/) and from two additional yeast two-hybrid datasets [15], [16] that are absent from this database. We focused on yeast two-hybrid datasets because the technique detects an interaction with minimal influence from endogenous environments, e.g. a fly cell. We assume that two proteins do not exhibit a PP interaction if both proteins were assayed and no interaction was found. To create a multi-species PP interaction network, we used the orthology mappings generated by InParanoid v1.35 [17] (non-default parameters: score cutoff 10, in-paralog confidence cutoff 0.025, sequence overlap cutoff 0.2) when run with protein sequences obtained from the InParanoid dataset from June, 2006 (http://inparanoid.sbc.su.se/cgi-bin/index.cgi). Comparisons with hand-curated orthologies for a subset of genes indicated that our parameter settings produced orthology mappings with minimal false positive results (data not shown). The names of the genes/proteins described in the datasets were updated to the names used in WormBase release WS180.

### Derivation of attributes for use in the logistic regression

The co-expression attribute *Exp*(*g*, *g*′) is the *P* value derived for the Pearson correlation of genes *g* and *g*′ across all microarray hybridizations (conditions) relative to the empirically estimated probability distribution of correlation for all gene pairs (i.e. a fitted normal). Figure S4 establishes the need for this estimation due to the lack of fit to standard models of a correlation distribution. Correlations greater than 0.35 are statistically significant (*P*≤0.05) according to the estimated distribution. The co-phenotype attribute *Ph*(*g*, *g*′) measures the statistical significance of the number of shared phenotypes between the two genes via a standard Fisher's exact test (*N* = the number of phenotypes observed for at least two genes). We defined the multi-species PP interaction network such that nodes represent *C. elegans* proteins and an edge exists between two proteins if they, or their orthologous proteins in a species considered here, exhibit a PP interaction according to the PP interaction dataset. The binary interaction attribute *I*(*g*, *g*′) indicates whether the proteins encoded by *g* and *g*′ exhibit a PP interaction in our multi-species PP interaction network (Figure 2A). Similarly, the common interactors attribute, *CI*(*g*, *g*′), considers the statistical significance of the observed number of common PP interactors of the proteins encoded by *g* and *g*′, in the multi-species PP interaction network (Figure 2B). Specifically, *CI*(*g*, *g*′) is assigned a *P* value derived from a one-tailed Fisher's exact test (*N* = the number of genes encoding proteins that are in the multi-species PP interaction network).

We defined a biological network called the PhEP network, where two genes *g* and *g*′ are connected by an edge if and only if the Pearson correlation of their gene expression exceeds 0.35, their gene products exhibit a PP interaction, or their orthologues (in any species considered here) exhibit a PP interaction (Figure 2C,D). For a given gene, we measured how surprising it is to witness the observed number of its neighbors (i.e. genes connected to it by one edge) in the PhEP network labeled with a specific phenotype identified by RNAi in *C. elegans*. This was measured using a one-tailed Fisher's exact test (*N* = the number of genes with some assigned phenotype). If the derived *P* value is less than or equal to 0.05 for *g* and *g*′ (Figure 2C), we assign a value of 1 to a categorical variable *N*(*g*, *g*′), and 0 otherwise. Similarly, if *g* and *g*′ exhibit a phenotype that is also enriched in both their neighborhoods in the PhEP network (Figure 2D), we assigned a value of 1 to a categorical variable *NPh*(*g*, *g*′), and 0 otherwise.

Missing values for any of the derived attributes (due to missing values in the underlying datasets) were replaced with the expected value (i.e. the sample mean) of the attribute before training.

### Model specification, training and cross-validation

The logistic regression model is of the form:
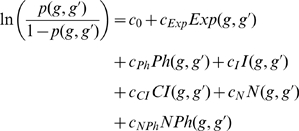
where *p*(*g*, *g*′) is the probability of a genetic interaction between genes *g* and *g*′, *c*_0_ is the learned intercept term of the model, *c_Exp_*, *c_Ph_*, *c_I_*, *c_CI_*, *c_N_*, *c_NPh_* are the learned coefficients for the attributes, and *Exp*(*g*, *g*′), *Ph*(*g*, *g*′), *I*(*g*, *g*′), *CI*(*g*, *g*′), *N*(*g*, *g*′) and *NPh*(*g*, *g*′) are the attribute values for *g* and *g*′.

To select the optimal logistic regression model in the context of our learning set and attributes, we assessed models defined by different attribute combinations and trained with different positive∶negative weight ratios. Specifically, we trained models using each of the following weight ratios: 1∶1, 1∶2, 1∶5, 1∶10 and 1∶100. If negative examples are weighted more heavily, prediction errors on these examples result in greater penalties, and model coefficients are fitted accordingly. Using each weight ratio, we trained the models defined by all non-empty subsets of the attributes (in total, 2^6^−1 = 63 models), with each of five different folds of the learning set to avoid learning set bias. In training each model with the iterative weighted least squares algorithm [61], we assume that the initial fit estimated from the weighted data is reasonably close to the optimal fit, and thus assume that the algorithm converges to the optimal fit (with the default tolerance and at most 50 iterations). For each fold, we define the optimal model as the model that yielded the lowest Akaike's Information Criterion (AIC), a measure that considers both fit to the data and complexity of the model. Any weight ratio that did not yield the same optimal model for all five folds was eliminated from consideration. For each remaining weight ratio, we computed the mean AIC of the optimal model (across the folds). The 1∶2 weight ratio yielded the lowest mean AIC and we thus selected this ratio and the corresponding optimal model to define our genetic interaction predictor. Therefore, within the scope of logistic regression models defined by our attributes and trained with our learning set and tested weight ratios, the full model that uses all six attributes was found to be optimal based on our convergence assumptions and the AIC (Table S7).

Leave-one-out cross-validation of the full model was performed to obtain true and false positive rates for “unseen” data (Figure S1). The final predictor was trained on the full learning set using the tuned weighting and all six attributes. If a pair of genes has a prediction score ≥0.85, the two genes are predicted to genetically interact.

Logistic regression is a technique that does not take into account the obvious dependencies between the attributes. To test the strength of dependencies between attributes we experimented with graphical models, specifically by using a software package for learning Bayesian networks (i.e. the deal package v1.2-30) [62]. The learning set used to train the logistic model was also used to train a Bayesian network. The resulting network exhibits several dependencies between the attributes (Figure S5), many of which are expected since some attributes are derived from the same underlying datasets. Although predictive accuracy might be improved if these attribute dependencies were accounted for, doing so would require a more sophisticated predictive model that relies on an abundance of data to accurately quantify the dependencies. Due to the paucity of attribute data for some genes (e.g. a gene may only have data for the *Exp* and *N* attributes), such a predictive model trained with the current datasets would not necessarily be advantageous over a simpler model (such as a logistic regression model).

### Predictions from other genetic interaction predictors

The functional interactions predicted by the Lee *et al.* method were obtained from the WormNet v1 core set [9]. The genetic interactions predicted by the Zhong and Sternberg method were downloaded in June, 2006 [10]. The names of the genes in these prediction datasets were updated to the names used in WormBase release WS180.

### Quantifying the information available for a gene

In quantifying the information available for a gene, we took into account the usefulness of particular types of data for the prediction of genetic interactions. Specifically, if there is sufficient data to compute the value of a predictive attribute (e.g. *Exp*) for any pair involving a particular gene, the usefulness of the value is quantified by the magnitude of the weight of the attribute in the predictive model (e.g. |*c_Exp_*|). The total quantity of information available for a gene is thus defined as the sum of the magnitudes of weights corresponding to attributes for which values can be computed. The quantities were scaled to be in [0,1] via division by the maximum quantity achievable. The subsequent relative quantities allow for comparisons between predictors that use different attributes (see Figure S3).

### Analysis of the predicted genetic interaction network with pathway annotations

The biological validity of the predicted genetic interaction network was assessed *in silico* by computing the shortest path distance between genes annotated to the same pathway. We defined the predicted network such that a node exists for each *C. elegans* gene and an edge exists between two genes if they are predicted to genetically interact. We also defined 100K randomized networks such that each randomized network is identical to the predicted network, except that the nodes are assigned a random permutation of the gene labels. *C. elegans* pathway annotations derived from human were obtained from KEGG release 44 (http://www.genome.jp/kegg/) and signaling pathway annotations derived directly from *C. elegans* were obtained from [63] (Table S3). Using the predicted network and each randomized network, the shortest path distance (i.e. the minimum number of edges to traverse in a given network to get from one gene to the other) was computed for every pairing of genes annotated to the same pathway. For each network, we subsequently computed *d_i_*, the number of pathway gene pairs with shortest path distance = *i*, for *i* = 1,2. *d*_1_ represents the number of within-pathway interactions based on the given set of pathway annotations (Figure 3A). *d*_2_ represents the number of pathway gene pairs that are not connected directly, but share ≥1 neighbor in the network, suggesting within- or between-pathway interactions (Figure 3B). Let *d_i_*_,*pred*_ represent *d_i_* of the predicted network. The significance of *d_i_*_,*pred*_ was estimated with a permutation *P* value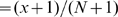 [64], where *x* is the number of randomized networks with *d_i_*≥*d_i_*_,*pred*_, and *N* is the total number of randomized networks. We further examined pathway gene pairs with shortest path distance = 2 in the predicted network. Specifically, we computed the percentage of these pairs that satisfy the following criterion: the given pair has ≥1 shared neighbor that is not annotated to any of the pathways associated with either member of the pair. The pairs that satisfy this criterion likely exhibit predicted within-pathway interactions with an unknown member of the pathway of the pair, or predicted between-pathway interactions.

### Nematode strains

Nematodes were grown on nematode growth media (NGM; Brenner, 1974) at 20°C. Bristol strain N2 animals were used as wild-type animals. Nematode strains containing the following alleles were retrieved from the *Caenorhabditis Genetic Center* (CGC), which is funded by the NIH National Center for Research Resources (NCRR): *rrf-1 (pk1417)*, *ppw-1 (pk2505)*, *dyb-1 (cx36)*, *unc-89 (e1460)*, *unc-89 (st85)*, *unc-89 (ok1116)*, *unc-89 (ok1659)*, *unc-96 (su151)*, *tra-4 (ok1636)*, *mel-11 (sb56)*, *trp-2 (gk298)* (WormBase: WBGene00006615), *smo-1 (ok359)* (WormBase: WBGene00004888), *aspm-1 (ok1208)*, *lin-36 (n766)* (WormBase: WBGene00003021), *F42G8.10 (ok1199)* (WormBase: WBGene00018361), *tag-163 (ok644)* (WormBase: WBGene00006508), *F54D5.4 (ok2046)* (WormBase: WBGene00010050), *dys-1 (cx18)*, *hlh-1 (cc561)* (see Table S8).

### RNAi and drug treatment

Blebbistatin (100 µM) and ML-7 (50 µM) were incorporated in NGM agar before plate pouring. The drug-containing plates were used throughout RNAi treatment. The pL4440-dest-*gdi-1* construct, used to submit animals to RNAi against *gdi-1*, was kindly provided by Dr Marc Vidal, Dana-Farber Cancer Institute. The pL4440-dest-*egfp* construct was generated as described previously [65]. These constructs were transformed into HT115 (DE3) strains [66] and the animals were submitted to RNAi treatment as previously described [67]. To score the sterility phenotype (Ste), synchronized L1 larvae were fed RNAi-expressing bacteria for 72h at 18°C. Three young adults were then transferred to fresh plates seeded with RNAi-expressing bacteria and they were allowed to lay eggs for 48h at 18°C. The progeny were counted and sterility was measured as detailed in the Epistasis statistics section. The penetrances of the endomitotic oocyte (Emo) and gonad morphogenesis defect (Gon) phenotypes were scored after DAPI staining the RNAi-treated animals fixed with methanol. Emo and Gon phenotypes were scored by fluorescence microscopy using a Leica DM5500 microscope equipped with a 63× oil-immersion objective and using regular sets of filters for excitation at an ultra-violet wavelength. An animal was considered as expressing the Emo phenotype if at least one endomitotic oocyte was present in the gonad. An animal was considered as expressing the Gon phenotype if its gonad was significantly shorter than gonads observed in N2 animals. The position of the gonad turn with respect to the anterior and posterior intestine nuclei was used to measure the relative length of a gonad. Muscle degeneration was observed in methanol-fixed nematodes upon polarized-light illumination, using a Leica DM5500 microscope equipped with a 100× oil-immersion objective. Only the centermost 20 cells of the two muscle quadrants facing the objective were observed to quantify the abnormal cells. Fluorescent microscopy pictures were captured using the Leica DFC350FX R2 camera and the Leica AF6000 software series. Polarized light microscopy pictures were captured from a Zeiss Axioimager Z1 equipped with a 63× oil-immersion objective and an Axiocam HRM camera controlled by the Axiovision software v4.5. The potential modulation of RNAi efficiency in the different backgrounds tested, and the relative contribution of balancers to identified genetic interactions, were examined to confirm the validity of our results (see Text S1 and Figure S6).

### Measurement of sheath cell contraction

Sheath cell contraction rates were scored in anesthetized animals (0.1% tricaine and 0.01% tetramisole in M9 buffer) as previously described [68]. Basal contractions were estimated by monitoring lateral sheath displacement [69] upon DIC illumination at room temperature, using a Leica DM5500 microscope equipped with a 63× oil-immersion objective.

### Epistasis statistics

Let *φ_x_* ∈ [0,1] represent the level of a particular phenotype expressed by genetic population *x*. Conversely, let 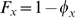 represent the “fitness” of *x* with respect to the phenotype, e.g. a maximal value of 1 indicates that the phenotypic defects are absent in all animals of type *x*. Let *φ_m_*, *φ_gdi-1_*, *φ_m_*_/*gdi-1*_ and *φ_wt_* represent the level of the phenotype expressed by animals with mutation(s) in (predicted interactor) gene *m*, wild-type animals submitted to *gdi-1(RNAi)*, *m*-mutant animals submitted to *gdi-1(RNAi)* and wild-type animals, respectively. Three different models were used to quantify epistatic effects through an epistasis coefficient *ε*. The models use values that have been normalized to wild-type levels, i.e. 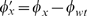 and 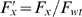. Under the minimum model [70]:
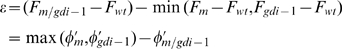
Under the additive model [25]:
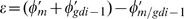
Under the multiplicative model [25]:
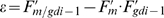
Within each model, a function of the phenotypic level expressed by the doubly-altered population (e.g. *φ_m_*_/*gdi-1*_) is compared to some expectation of the level, given what is known about the populations with the single-gene perturbations. This expectation is computed by a model-specific function, *f*(*φ_m_*, *φ_gdi-1_*, *φ_wt_*) ∈ [0,1]. For example, under the additive model, 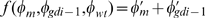 if 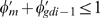, otherwise 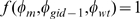.

If *ε*<0, there is a synergistic interaction between *m* and *gdi-1*. If *ε*>0, there is an antagonistic interaction. We also identified genes that, when mutated, specifically suppress the phenotypic effects of *gdi-1(RNAi)* (observed in wild-type animals). This was achieved by statistically testing if 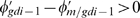. See Text S1 for details regarding all statistical tests performed, including details about our normality assumption (Figure S7).

The Ste level expressed by a genetic population *x* was defined as 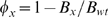, where *B_x_* and *B_wt_* are the brood size measurements for *x* and wild-type animals, respectively. The Gon and Emo levels were defined as 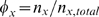, where *n_x_* is the number of *x* animals observed to have the phenotype and *n_x,total_* is the total number of *x* animals examined.

### Statistic for the suppression of muscle degeneration

Let 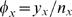 represent the level of muscle degeneration expressed by an animal in genetic population *x*, where *y_x_* is the number of abnormal muscle cells and *n_x_* is the total number of muscle cells observed in the animal. In each independent experiment, at least 20 animals were observed for each genetic population. We statistically tested the hypothesis that *φ_m/gdi-1_*<*φ_m_*, i.e. *gdi-1(RNAi)* treatment suppresses the muscle degeneration observed in *m*-mutant animals. Specifically, the hypothesis was tested using the Mann-Whitney test and a *P* value was obtained for each independent experiment. The *P* values were combined to compute an overall *P* value using the weighted-*Z* method [71] (*N* = 3). The weight of each independent experiment was the total number of animals observed (i.e. the number of *gdi-1(RNAi)* treated *m*-mutant animals observed plus the number of control-treated *m*-mutant animals observed).

## Supporting Information

Text S1 Supporting Methods.
(0.05 MB DOC)

Figure S1 Receiver-operating-characteristic curve of the genetic interaction predictor. The error rates were estimated with leave-one-out cross-validation. The threshold associated with each point (i.e. a pair of rates) is indicated in red text. Only the portion of the curve with the smallest false positive rates is shown since, in practice, having fewer false positives instead of greater sensitivity is more important for laborious experimental validation.
(0.08 MB TIF)

Figure S2 Comparison of genome-wide genetic interactions predicted by different approaches. (A) Venn diagrams of predicted interactions from Zhong and Sternberg [10], Lee *et al.* [9] and this study. Left, interactions between any *C. elegans* genes. Right, interactions between *C. elegans* genes with human orthologues. Our approach predicts many novel interactions and about 85% of them are between *C. elegans* genes without human orthologues. (B) Comparison of the mean number of predicted interactions per gene and the percentage of genes with predicted interactions (i.e. the percentage of the genome covered by the set of predicted interactions), between two studies. The comparisons are made in the context of mental retardation and synaptic plasticity (MRSP) genes only and in the genome-wide context (GW). (C) Comparison of the number of human genes whose *C. elegans* orthologues have predicted interactions, stratified by gene characterization index (see Text S1). Our approach predicts novel interactions for genes orthologous to poorly-characterized human genes.
(0.42 MB TIF)

Figure S3 The relationship between the quantity of information available for a gene and the number of predicted genetic interactions. The quantity of information available for a gene is a measure that takes into account the fact that some gene pair attributes are more informative than others for predicting genetic interactions. See the Methods for the computation of the total quantity of information for each gene. MRSP: mental retardation and synaptic plasticity; ZS: Zhong and Sternberg [10]. (A) The total quantity of information available for MRSP genes with the ZS approach and with our approach. The three sets of boxplots correspond to MRSP genes with predicted interactions in this study only, in the ZS study only and in neither study, respectively. (B) Types and total quantity of information available for MRSP genes with the ZS approach and with our approach. Each column corresponds to a gene and a black entry indicates that there is information for the gene of the type specified (to the left) by the row (except for the row labeled “Total quantity of information”). The ZS approach separates the information from three organisms: *Saccharomyces cerevisiae* (*Sc*), *Drosophila melanogaster* (*Dm*) and *Caenorhabditis elegans* (*Ce*). The information types (i.e. attributes) of this study are described in the Results and Methods. For each approach, there is also a row indicating the total quantity of information (scaled between 0 and 1), where white and black indicate zero and maximal information, respectively. The heatmap in the middle illustrates the number of interactions predicted for each gene by the different approaches, where a greater intensity of red corresponds to a greater number. *gdi-1* is highlighted in green.
(1.22 MB TIF)

Figure S4 Different methods for estimating the *P* value associated with a Pearson correlation value measuring the coexpression of two genes in the Kim *et al.* dataset [14]. The grey bars indicate the empirical *P* values associated with bins of correlation values. The *t*-distribution (blue line) and Fisher's *Z* transform (red line) methods do not produce *P* values that match the empirical trend closely. In contrast, the fitted normal distribution approximates the empirical distribution well (green line).
(0.14 MB TIF)

Figure S5 The dependencies between the predictive gene pair attributes as defined by a learned Bayesian network. See the Methods for how the Bayesian network was derived.
(0.13 MB TIF)

Figure S6 The interaction of *gdi-1* with unbalanced heterozygotes of *aspm-1(ok1208)*. The mean penetrance/expressivity of the Emo phenotype in wild-type (*wt*) or unbalanced *aspm-1(ok1208)* heterozygotes (*aspm-1(ok1208)* +/−), submitted to either *egfp* or *gdi-1* RNAi, is shown. The error bars correspond to ± one standard error over three independent experiments. (*) indicates a statistical difference between *wt* and *aspm-1(ok1208)* +/− animals submitted to *gdi-1(RNAi)* (*P*≤0.05, see Methods).
(0.08 MB TIF)

Figure S7 Validity of the normality assumption for the application of Student's *t*-tests to phenotype measurement data. The bars represent the empirical distribution of scaled phenotype values induced by *gdi-1(RNAi)* treatment (see Text S1). Each red line is a fitted normal distribution.
(0.11 MB TIF)

Table S1 Genetic interactions hand-curated from the literature.
(0.05 MB XLS)

Table S2 Performance of genetic interaction predictors.
(0.02 MB XLS)

Table S3 Signaling pathway genes curated from the *C. elegans* literature.
(0.05 MB XLS)

Table S4 Curated set of mental retardation and synaptic plasticity genes and their *C. elegans* orthologues (204 genes).
(0.04 MB XLS)

Table S5 Epistasis coefficients of experimentally tested genetic interactions.
(0.03 MB XLS)

Table S6 Epistasis *P* values of experimentally tested genetic interactions.
(0.02 MB XLS)

Table S7 AIC values of 63 logistic regression models that use different combinations of the gene pair attributes.
(0.03 MB XLS)

Table S8 Genotypes of *C. elegans* strains used in this study.
(0.02 MB XLS)
