# Supplementary material for: Searching for Signaling Balance through the Identification of Genetic Interactors of the Rab Guanine-Nucleotide Dissociation Inhibitor gdi-1
Source: PLoS One. 2010 May 13;5(5):e10624. doi: 10.1371/journal.pone.0010624 (PMC2869356; doi:10.1371/journal.pone.0010624)
Supplement: Text S1 — Supporting Methods. (0.05 MB DOC) [file pone.0010624.s001.doc]

**SUPPORTING METHODS**

**Human orthologues of genes with predicted genetic interactions**

The set of predicted genetic interactions was assessed in terms of usefulness for improving the characterization of human genes. HomoloGene orthology (release 56, downloaded from ftp://ftp.ncbi.nih.gov/pub/HomoloGene/) was used to map *C. elegans* genes to human genes. Gene characterization indices of human genes were obtained from a previous study [1]. Figure S2C summarizes the results of this assessment.

**Controls for the validation of predicted genetic interactions using RNAi and balanced heterozygote strains**

In order to test whether the observed reduction of *gdi-1(RNAi)*-induced phenotypes in various mutant backgrounds was due to an overall reduction of RNAi efficiency (instead of an interaction), mutant animals expressing YOLK::GFP proteins were generated by mating mutant hermaphrodites with *sqt-1(sc103) II; bIs1 X* males. Efficiency of *egfp(RNAi)* treatment was estimated by counting animals without observable GFP emission using fluorescence microscopy. No difference in *egfp(RNAi)* efficiency was observed in *dyb-1 (cx36)*, *unc-89 (e1460)*, *unc-89 (st85)*, *unc-89 (ok1116)*, *unc-89 (ok1659)*, *unc-96 (su151)*, *tra-4 (ok1636) and aspm-1 (ok1208)* backgrounds (data not shown). This confirmed the genetic interactions between these genes and *gdi-1.*

Interactions with *gdi-1* were assessed through RNAi treatment of homozygotes for all alleles tested except for *aspm-1 (ok1208)*. Instead, *aspm-1 (ok1208)* heterozygotes were balanced with a hT2 chromosomal translocation between chromosomes I and III. In order to assess whether *gdi-1* also genetically interacts with unbalanced heterozygotes of *aspm-1(ok1208)*, *VC761* (*aspm-1(ok1208)I* / *hT2[bli-4(e937); let-?(q782)qsl48]*)animals were mated with N2 animals. L1 larvae without GFP expression in the pharynx (corresponding to unbalanced *aspm-1(ok1208)*) were isolated from the F1 population and submitted to RNAi against *gdi-1* or *egfp*. The Emo phenotype was then scored as detailed in the Methods. As shown in Figure S6, the *gdi-1(RNAi)*-induced Emo phenotype was significantly reduced in unbalanced *aspm-1(ok1208)* heterozygotes. This confirms the interaction between *gdi-1* and *aspm-1* (Figures 5A and 6).

**Epistasis statistics**

The statistical significance of genetic interactions with respect to the Gon and Emo phenotypes was determined as follows. Consider the putative interaction between a gene *m* and *gdi-1*. For some genes, multiple alleles were tested via different strains (e.g. *unc-89*). In these cases, epistasis statistics were computed for each individual strain. Due to day effects, the epistasis coefficient *εi* was computed independently for each day *i* on which the experiments were replicated. Let *wi* represent the total number of animals examined on day *i* (with respect to gene *m* and *gdi-1*). The weighted mean and variance of *ε* were computed using the *wi­* values as weights. Finally, these weighted statistics of *ε* were used to compute a *t-*statistic for testing the null hypothesis that *ε =* 0 (i.e. a two-side test) with (*N* – 1) degrees of freedom, where *N* is the number of days on which the experiments were replicated.

Similarly, the test for *gdi-1(RNAi)* suppressors was performed by computing a *t-*statistic based on the weighted mean and variance of *d = **gdi-1* – *φ**m/gdi-1*. Since the goal was to test the null hypothesis that *d* 0, a one-sided test was used to determine statistical significance. Figure 5 illustrates values of (-*d*) so that a reduction in phenotypic level is associated with a negative value (green bars). Analogous statistics were computed and plotted for the *egfp(RNAi)* observations (Figure 5, grey bars) for which we defined *d = **egfp* – *φ**m/egfp*.

For the Ste phenotype, each genetic population was quantified on different days yet also replicated within each day. The same day measurements were used to estimate the variance of each population and paired measurements across days were used to estimate the covariance of different populations. For day *i*, *εi* was computed from the mean brood sizes of the relevant genetic populations. The standard error of *εi* was estimated using a formula derived from error propagation rules [2]. For cases where the formula yielded an invalid standard error value (e.g. by taking the square root of a negative number), the delta method [3] was used to estimate the error. If this failed as well, across-day variance for each population was used in place of the within-day variance as input to the delta method. A *t-*statistic for *εi* was then computed and used to test the null hypotheses *εi* < 0 and *εi* > 0 (i.e. one-sided tests) with ν*i* = *Ni,*1 *+ Ni,*2 *+… + Ni,k* – *k* degrees of freedom, where *Ni*,*j* is the number of replicates on day *i* for population *j*,and *k* is the number of populations relevant to the computation of *εi*. For each null hypothesis, the derived *P* values, *Pi*, were combined to compute an overall *P* value using the weighted-*Z* method [4] with the ν*i* values used as weights. Consequently, we obtained one *P* value for the significance of an antagonistic interaction (*εi* > 0), and a second for the significance of a synergistic interaction (*εi* < 0).

We opted for two one-tailed tests instead of a single two-tailed test for Ste since combining *P* values that each test the (two-sided) null hypothesis that *εi* = 0 can be misleading. For example, if *P1* is low because *ε1* > 0 and *P2* is low because *ε2* < 0, the combined *P* valuewill likely be low as well even though the across-day average *ε* is likely close to zero. As a result, the significance of the interaction would be over-estimated. Alternatively, *Pi* values from one-sided tests of the same null hypothesis (e.g. *ε* < 0) can only be low if the respective *εi* values are extreme in a pre-specified direction (e.g. *ε* > 0). Therefore, the combined *P* value will reflect the overall significance of *ε* being extreme in that specific direction (e.g. *ε* > 0; antagonistic).

Similarly, the test for *gdi-1(RNAi)* suppressors in terms of Ste was performed by computing a *t-*statistic for *d = **gdi-1* – *φ**m/gdi-1*. Specifically, *di* was computed for each day *i* on which the experiment was repeated. The standard error of *di* was estimated with the same techniques used to estimate the standard error of *εi*. A *t-*statistic for *di* was then computed and used to test the null hypothesis *di* < 0 (i.e. a one-sided test) with ν*i* = *Ni,*1 *+ Ni,*2 *+… + Ni,k* – *k* degrees of freedom, where *Ni*,*j* is the number of replicates on day *i* for population *j*,and *k* is the number of populations relevant to the computation of *di*. Again, the derived *Pi* were combined to compute an overall *P* value using the weighted-*Z* method [4] with the ν*i* values used as weights. Figure 5 illustrates values of the weighted *Z*-score so that a reduction in phenotypic level is associated with a negative value (green bars), and the error bars correspond to the standard error of the *Z*-score. Analogous statistics were computed and plotted for the *egfp(RNAi)* observations (Figure 5, grey bars) for which we defined *d = **egfp* – *φ**m/egfp*.

Since the statistical tests were repeated for different genes/strains, the resulting *P* values were adjusted for multiple comparisons using the Benjamini and Hochberg method [5]. For all tests, a threshold of (adjusted *P*) ≤ 0.05 was used to determine statistical significance. All epistasis values and corresponding adjusted *P* values are provided in Tables S5 and S6, respectively.

The *t*-distribution was used throughout under the assumption that the errors (i.e. deviations of the measurements from the true values) are normally distributed. The validity of this assumption was assessed by considering the *gdi-1(RNAi)*-treated animals since there are roughly 30 measurements per phenotype for this genetic population whereas other populations have much fewer. To make the measurements comparable, we weighted the wild-type-normalized values to reflect the confidence in the measurement. The weight of a measurement was derived from the sample size used to obtain it (e.g. the number of worms examined for the phenotype on a particular day). Specifically, we defined the weight as (sample size)/(total sample size across days). Figure S7 illustrates the histogram of the final scaled measurements along with a fitted normal distribution for each phenotype. Although the true distributions are bounded below by zero, their shapes are generally normal as required. The bound discrepancy simply leads to more conservative *P* values. The *t*-test is also appropriate since the data are often obtained from few samples and the *t*-distribution is explicitly parameterized by sample size (via degrees of freedom) accordingly.

**REFERENCES**

1. Kemmer D, Podowski RM, Yusuf D, Brumm J, Cheung W, et al. (2008) Gene characterization index: assessing the depth of gene annotation. PLoS ONE 3: e1440.

2. Bevington PR (1969) Data Reduction and Error Analysis for the Physical Sciences. New York: McGraw-Hill.

3. Oehlert GW (1992) A note on the delta method. American Stat 46: 27-29.

4. Whitlock MC (2005) Combining probability from independent tests: the weighted Z-method is superior to Fisher's approach. J Evol Biol 18: 1368-1373.

5. Benjamini Y, Hochberg Y (1995) Controlling the false discovery rate: a practical and powerful approach to multiple testing. J R Stat Soc B 57: 289-300.
