# Supplementary figures and images for: Searching for Signaling Balance through the Identification of Genetic Interactors of the Rab Guanine-Nucleotide Dissociation Inhibitor gdi-1
Source: PLoS One. 2010 May 13;5(5):e10624. doi: 10.1371/journal.pone.0010624 (PMC2869356; doi:10.1371/journal.pone.0010624)

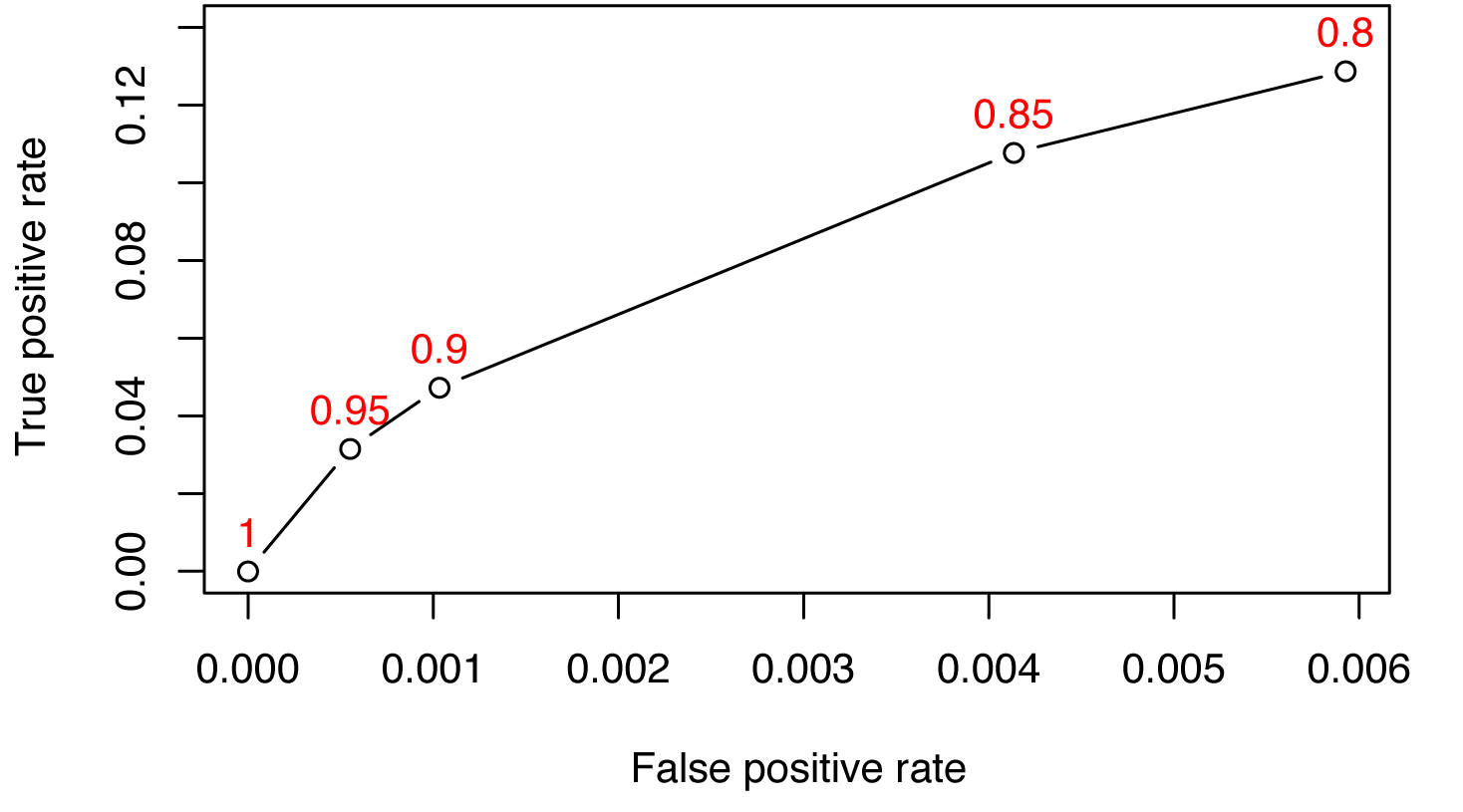

Supplement: Figure S1 — Receiver-operating-characteristic curve of the genetic interaction predictor. The error rates were estimated with leave-one-out cross-validation. The threshold associated with each point (i.e. a pair of rates) is indicated in red text. Only the portion of the curve with the smallest false positive rates is shown since, in practice, having fewer false positives instead of greater sensitivity is more important for laborious experimental validation. (0.08 MB TIF) [file pone.0010624.s002.tif]

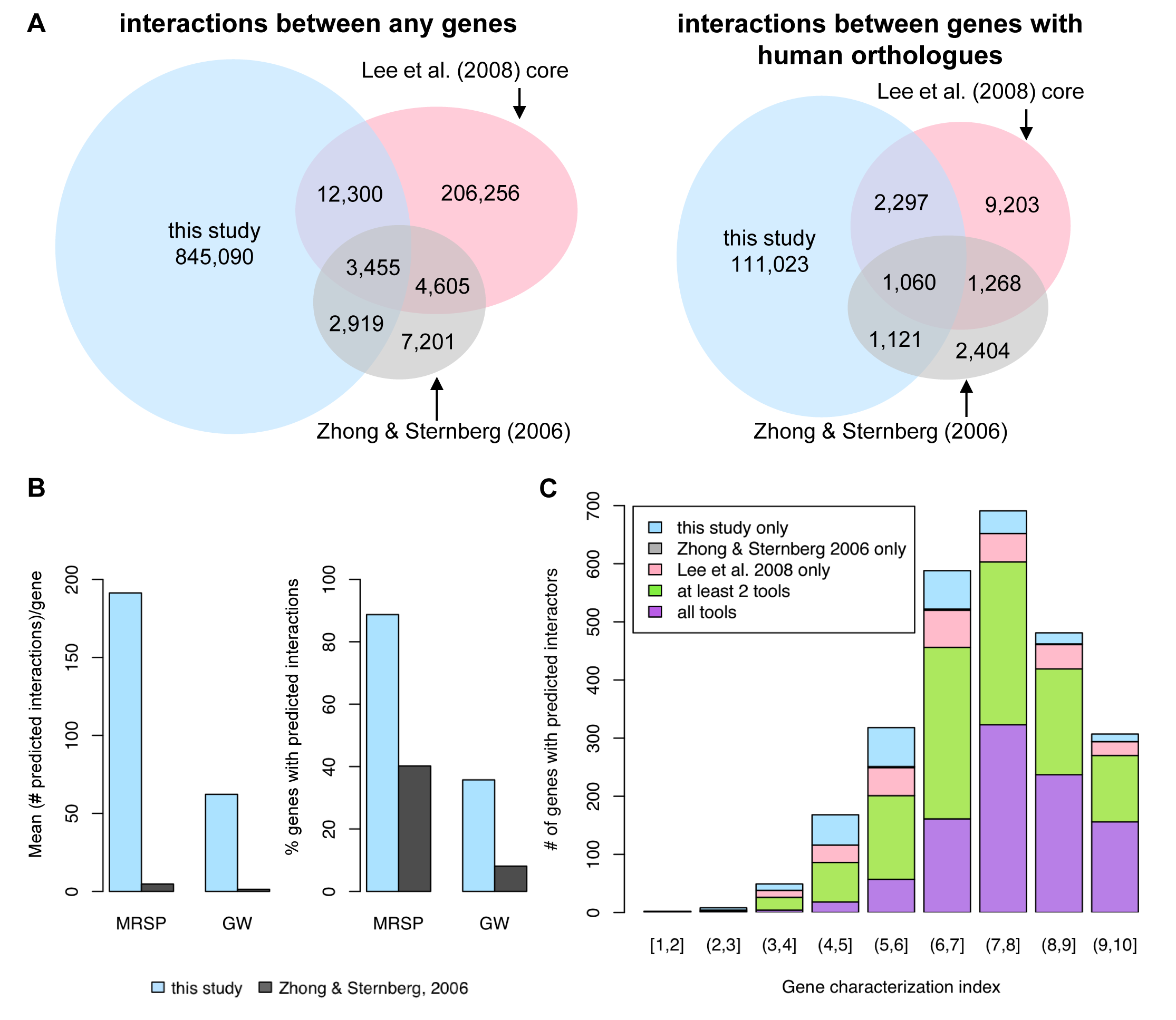

Supplement: Figure S2 — Comparison of genome-wide genetic interactions predicted by different approaches. (A) Venn diagrams of predicted interactions from Zhong and Sternberg [10], Lee et al. [9] and this study. Left, interactions between any C. elegans genes. Right, interactions between C. elegans genes with human orthologues. Our approach predicts many novel interactions and about 85% of them are between C. elegans genes without human orthologues. (B) Comparison of the mean number of predicted interactions per gene and the percentage of genes with predicted interactions (i.e. the percentage of the genome covered by the set of predicted interactions), between two studies. The comparisons are made in the context of mental retardation and synaptic plasticity (MRSP) genes only and in the genome-wide context (GW). (C) Comparison of the number of human genes whose C. elegans orthologues have predicted interactions, stratified by gene characterization index (see Text S1). Our approach predicts novel interactions for genes orthologous to poorly-characterized human genes. (0.42 MB TIF) [file pone.0010624.s003.tif]

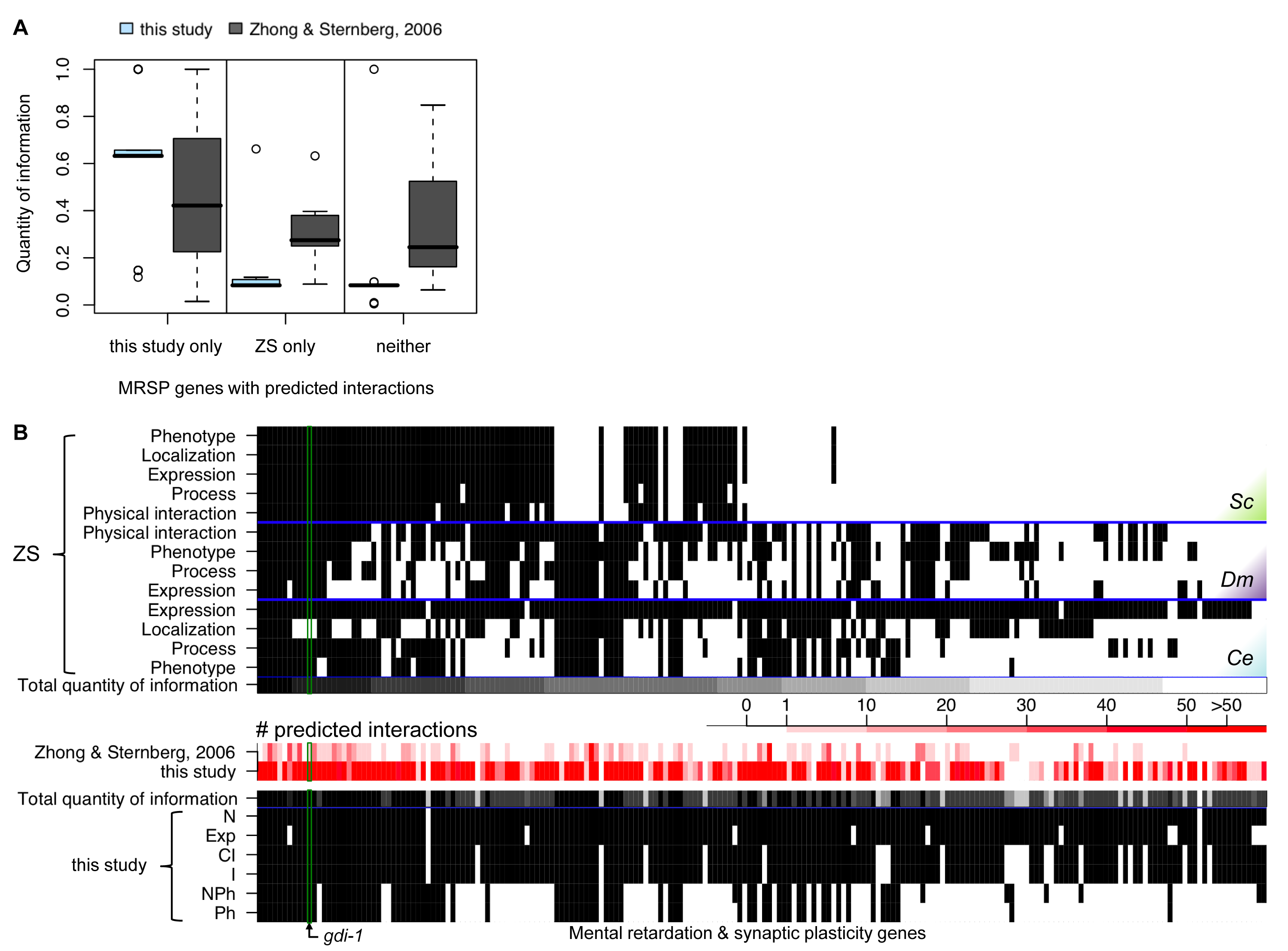

Supplement: Figure S3 — The relationship between the quantity of information available for a gene and the number of predicted genetic interactions. The quantity of information available for a gene is a measure that takes into account the fact that some gene pair attributes are more informative than others for predicting genetic interactions. See the Methods for the computation of the total quantity of information for each gene. MRSP: mental retardation and synaptic plasticity; ZS: Zhong and Sternberg [10]. (A) The total quantity of information available for MRSP genes with the ZS approach and with our approach. The three sets of boxplots correspond to MRSP genes with predicted interactions in this study only, in the ZS study only and in neither study, respectively. (B) Types and total quantity of information available for MRSP genes with the ZS approach and with our approach. Each column corresponds to a gene and a black entry indicates that there is information for the gene of the type specified (to the left) by the row (except for the row labeled “Total quantity of information”). The ZS approach separates the information from three organisms: Saccharomyces cerevisiae (Sc), Drosophila melanogaster (Dm) and Caenorhabditis elegans (Ce). The information types (i.e. attributes) of this study are described in the Results and Methods. For each approach, there is also a row indicating the total quantity of information (scaled between 0 and 1), where white and black indicate zero and maximal information, respectively. The heatmap in the middle illustrates the number of interactions predicted for each gene by the different approaches, where a greater intensity of red corresponds to a greater number. gdi-1 is highlighted in green. (1.22 MB TIF) [file pone.0010624.s004.tif]

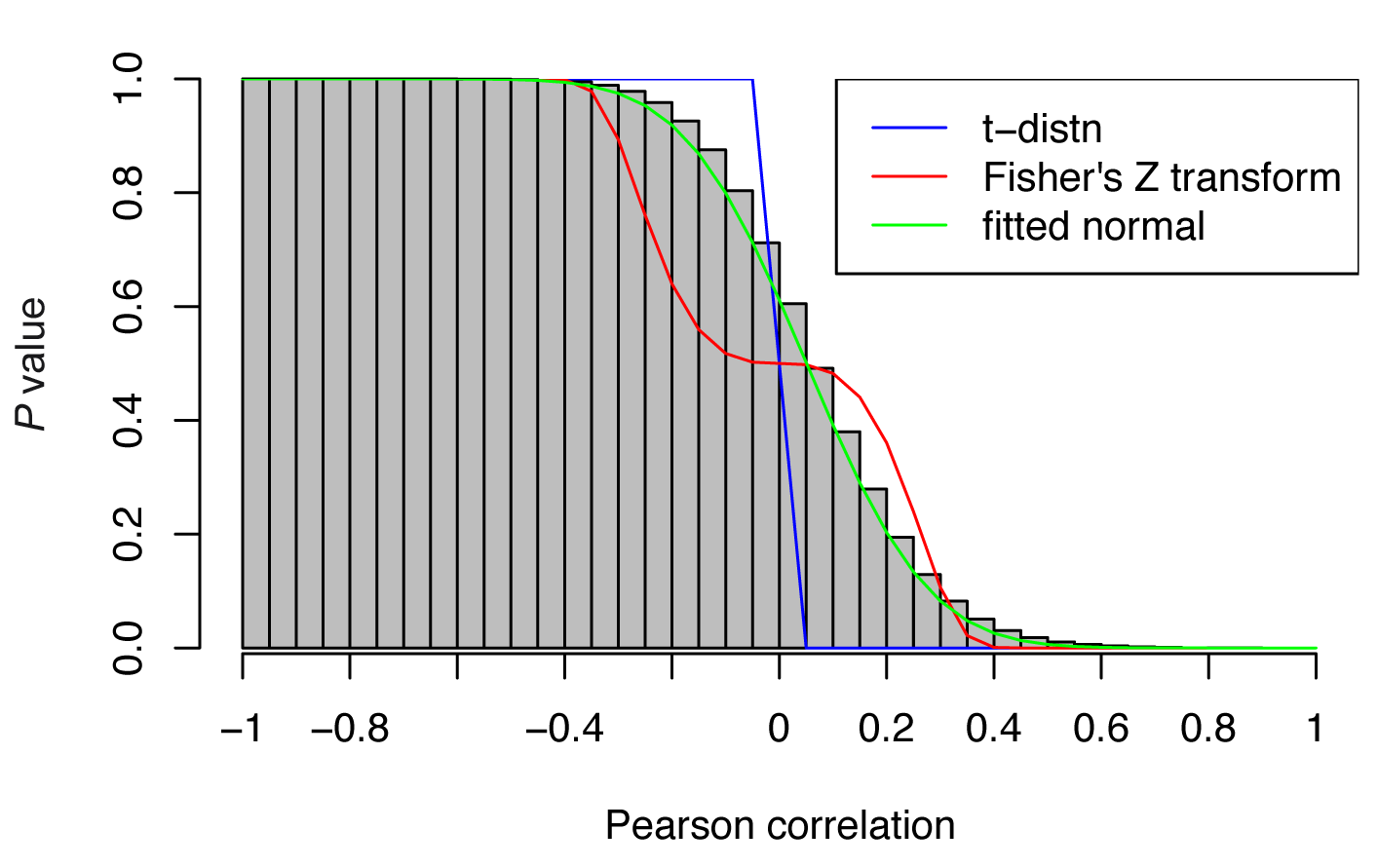

Supplement: Figure S4 — Different methods for estimating the P value associated with a Pearson correlation value measuring the coexpression of two genes in the Kim et al. dataset [14]. The grey bars indicate the empirical P values associated with bins of correlation values. The t-distribution (blue line) and Fisher's Z transform (red line) methods do not produce P values that match the empirical trend closely. In contrast, the fitted normal distribution approximates the empirical distribution well (green line). (0.14 MB TIF) [file pone.0010624.s005.tif]

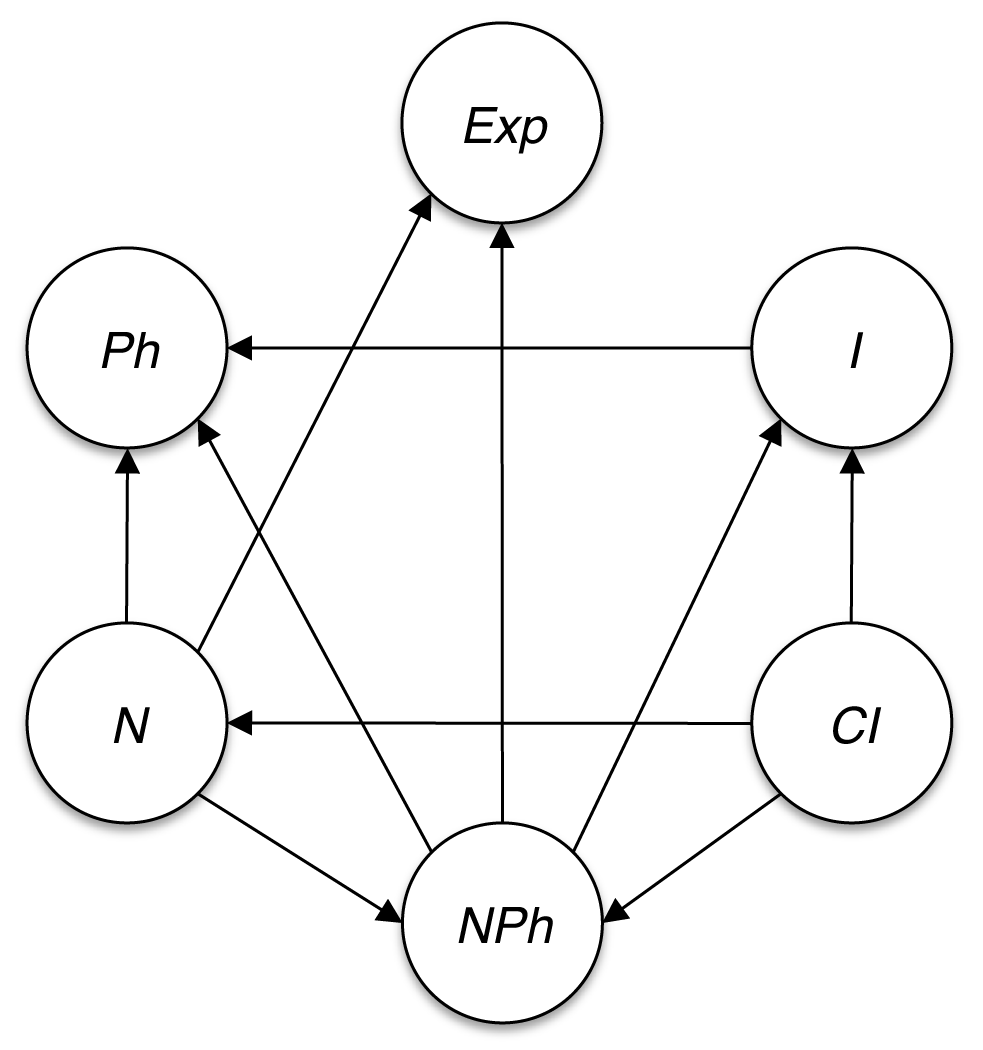

Supplement: Figure S5 — The dependencies between the predictive gene pair attributes as defined by a learned Bayesian network. See the Methods for how the Bayesian network was derived. (0.13 MB TIF) [file pone.0010624.s006.tif]

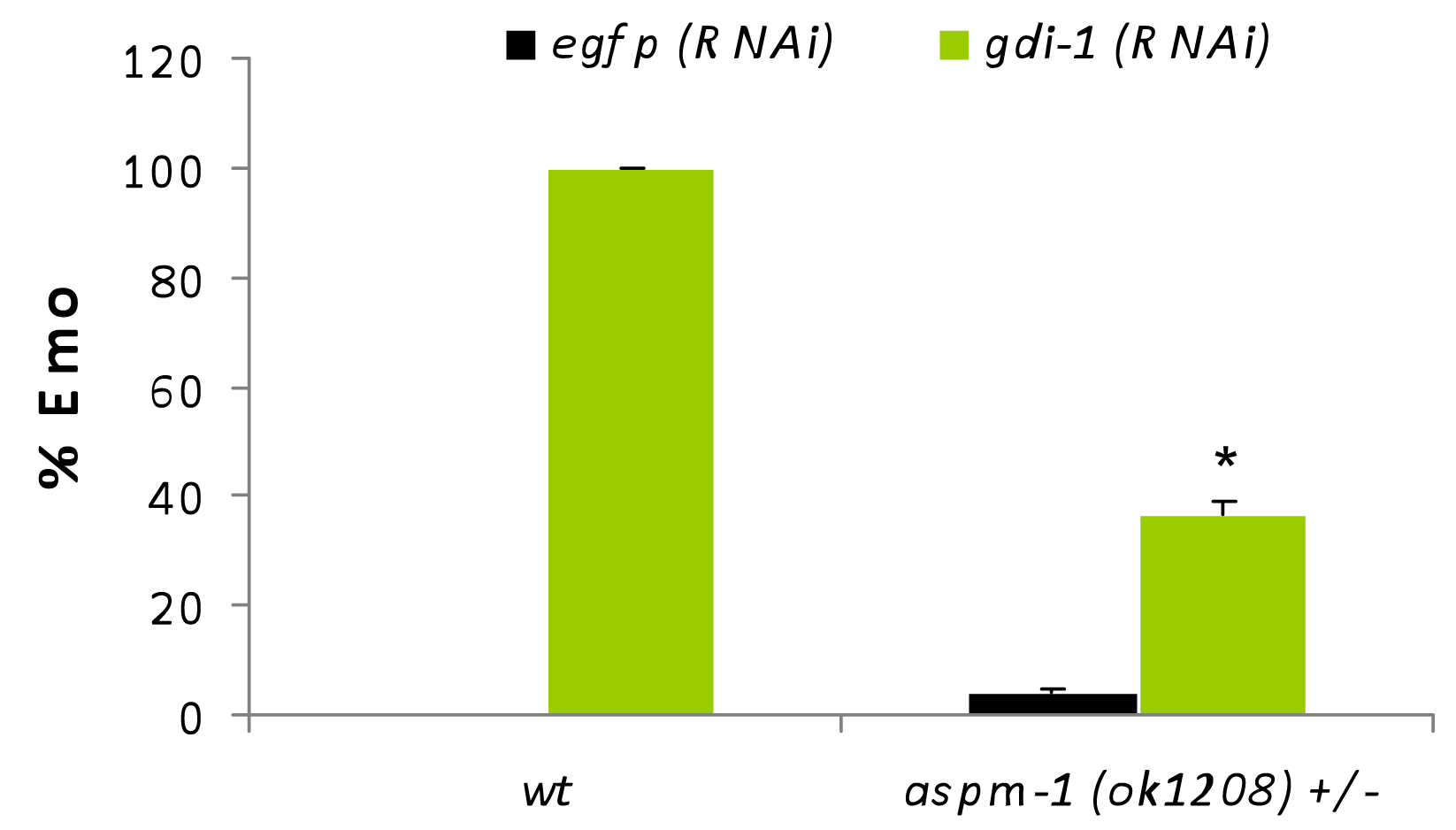

Supplement: Figure S6 — The interaction of gdi-1 with unbalanced heterozygotes of aspm-1(ok1208). The mean penetrance/expressivity of the Emo phenotype in wild-type (wt) or unbalanced aspm-1(ok1208) heterozygotes (aspm-1(ok1208) +/−), submitted to either egfp or gdi-1 RNAi, is shown. The error bars correspond to ± one standard error over three independent experiments. (*) indicates a statistical difference between wt and aspm-1(ok1208) +/− animals submitted to gdi-1(RNAi) (P≤0.05, see Methods). (0.08 MB TIF) [file pone.0010624.s007.tif]

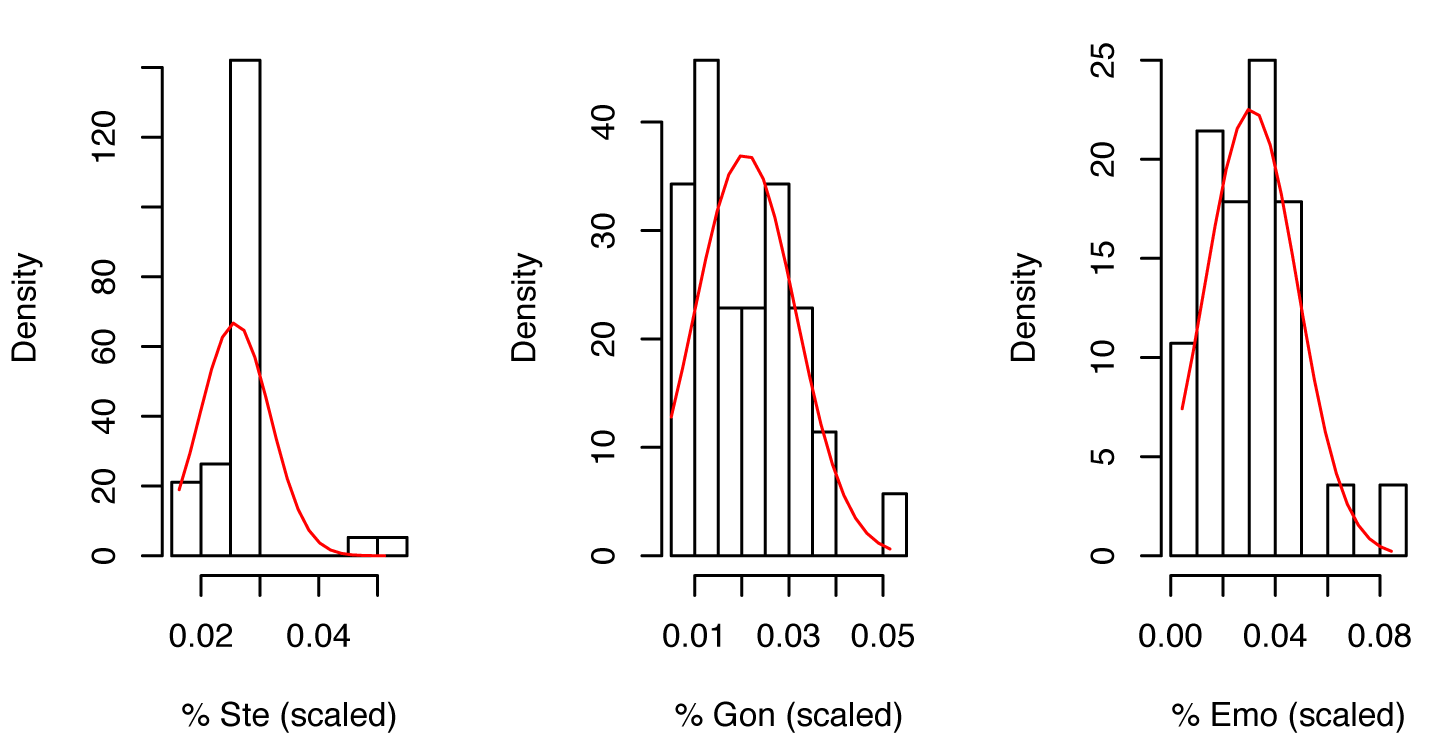

Supplement: Figure S7 — Validity of the normality assumption for the application of Student's t-tests to phenotype measurement data. The bars represent the empirical distribution of scaled phenotype values induced by gdi-1(RNAi) treatment (see Text S1). Each red line is a fitted normal distribution. (0.11 MB TIF) [file pone.0010624.s008.tif]
